# The Genomic Distribution and Function of Histone Variant HTZ-1 during *C. elegans* Embryogenesis

**DOI:** 10.1371/journal.pgen.1000187

**Published:** 2008-09-12

**Authors:** Christina M. Whittle, Karissa N. McClinic, Sevinc Ercan, Xinmin Zhang, Roland D. Green, William G. Kelly, Jason D. Lieb

**Affiliations:** 1 Department of Biology, University of North Carolina at Chapel Hill, Chapel Hill, North Carolina, United States of America; 2 Carolina Center for the Genome Sciences, University of North Carolina at Chapel Hill, Chapel Hill, North Carolina, United States of America; 3 Department of Biology, Emory University, Atlanta, Georgia, United States of America; 4 Genetics and Molecular Biology Program, Emory University, Atlanta, Georgia, United States of America; 5 Nimblegen Systems, Inc., Madison, Wisconsin, United States of America; European Molecular Biology Laboratory, Germany

## Abstract

In all eukaryotes, histone variants are incorporated into a subset of nucleosomes to create functionally specialized regions of chromatin. One such variant, H2A.Z, replaces histone H2A and is required for development and viability in all animals tested to date. However, the function of H2A.Z in development remains unclear. Here, we use ChIP-chip, genetic mutation, RNAi, and immunofluorescence microscopy to interrogate the function of H2A.Z (HTZ-1) during embryogenesis in *Caenorhabditis elegans*, a key model of metazoan development. We find that HTZ-1 is expressed in every cell of the developing embryo and is essential for normal development. The sites of HTZ-1 incorporation during embryogenesis reveal a genome wrought by developmental processes. HTZ-1 is incorporated upstream of 23% of *C. elegans* genes. While these genes tend to be required for development and occupied by RNA polymerase II, HTZ-1 incorporation does not specify a stereotypic transcription program. The data also provide evidence for unexpectedly widespread independent regulation of genes within operons during development; in 37% of operons, HTZ-1 is incorporated upstream of internally encoded genes. Fewer sites of HTZ-1 incorporation occur on the X chromosome relative to autosomes, which our data suggest is due to a paucity of developmentally important genes on X, rather than a direct function for HTZ-1 in dosage compensation. Our experiments indicate that HTZ-1 functions in establishing or maintaining an essential chromatin state at promoters regulated dynamically during *C. elegans* embryogenesis.

## Author Summary

To fit within a cell's nucleus, DNA is wrapped around protein spools composed of the histones H3, H4, H2A, and H2B. One spool and the DNA wrapped around it are called a nucleosome, and all of the packaged DNA in a cell's nucleus is collectively called “chromatin.” Chromatin is important because it modulates access to information encoded in the underlying DNA. Spools with specialized functions can be created by replacing a typical histone component with a variant version of the histone protein. Here, we examine the distribution and function of the *C. elegans* histone H2A variant H2A.Z (called HTZ-1) during development. We demonstrate that HTZ-1 is required for proper development, and that embryos are dependent on a contribution of HTZ-1 from their mothers for survival. We mapped the location of HTZ-1 incorporation genome-wide and found that HTZ-1 binds upstream of 23% of genes, which tend to be genes that are essential for development and occupied by RNA polymerase. Fewer sites of HTZ-1 incorporation were found on the X chromosome, probably due to an under-representation of essential genes on X rather than a direct role for HTZ-1 in X-chromosome dosage compensation. Our study reveals how the genome is remodeled by HTZ-1 to allow the proper regulation of genes critical for development.

## Introduction

In genomes ranging from protozoa to humans, specialized regions of chromatin are created by the local incorporation of variant histones into nucleosomes. The histone H2A variant H2A.Z is one such highly conserved variant, though the biophysical and biological function of H2A.Z incorporation into chromatin remains unresolved.

Evidence from *Tetrahymena* suggests a function for H2A.Z in transcriptional activation due to its localization to the transcriptionally active macronucleus [1]–[3]. This function is consistent with genome-wide studies of Htz1 occupancy in *S. cerevisiae* (hereafter “yeast”), which revealed Htz1 incorporation flanking a nucleosome-free region upstream of most genes. It has been hypothesized that H2A.Z-containing nucleosomes may contribute to transcriptional activation by being less stable than H2A-containing nucleosomes [4]–[6]. However, others have reported that H2A.Z-containing nucleosomes are in fact slightly more stable than canonical nucleosomes [7]–[9]. This seeming contradiction may have been resolved by studies examining H2A.Z in combination with the histone H3 variant H3.3. In combination with histone H3, H2A.Z nucleosomes were at least as stable as H2A nucleosomes, but the combination of H2A.Z and H3.3 results in highly unstable nucleosomes [4]. This instability in conjunction with H3.3 could facilitate timely and efficient gene activation. Indeed, in yeast cells lacking H2A.Z, the activation of genes in response to heat shock or galactose is delayed, and recruitment of RNA polymerase II and TATA-binding protein to responsive promoters is diminished [10],[11]. H2A.Z is also required for a form of “transcriptional memory” in yeast, in which recently transcribed chromatin is retained at the nuclear membrane to allow rapid re-activation of the gene [12]. Recent high-resolution mapping of H2A.Z in human cells also revealed a positive correlation between H2A.Z occupancy and transcription, providing additional support for an H2A.Z function in transcriptional activation [13].

Despite the wealth of evidence for a positive association between H2A.Z and transcription, other genetic and cytological evidence suggests that H2A.Z also functions in gene silencing. The functional homolog of H2A.Z in *Drosophila*, H2Avd, is localized to both euchromatin and heterochromatin on polytene chromosomes, including the heterochromatic chromocenter [14],[15]. By genetic criteria, H2Avd is considered to have a repressive function. H2Avd mutations are enhancers of Polycomb mutant phenotypes, suppressors of Trithorax group mutant phenotypes, and suppressors of position-effect variegation [16]. Further evidence for a repressive function is found in mice, where H2A.Z promotes heterochromatin protein HP1α binding and co-localizes with HP1 at pericentric heterochromatin [17],[18]. In mammalian cells, mono-ubiquitylation of the H2A.Z C-terminus may distinguish “repressive H2A.Z” from “activating H2A.Z”, particularly on the silent X chromosome [19]. Even within the yeast literature, there are conflicting conclusions regarding correlation with transcriptional activity and RNA Polymerase II. One study found no correlation between Htz1 occupancy and transcription rate of the downstream gene [11], while others reported an inverse correlation with transcription rate [20]–[22].

The resolution of these apparently contradictory activating and silencing functions could be explained by a requirement for H2A.Z in regulating the precise timing and kinetics transcription, rather than simply promoting an “on” or “off” transcriptional state. This potentiation of transcription would be especially critical during periods of dynamic transcriptional regulation, such as occurs in development and environmental responses. There is growing evidence for this hypothesis. In *C. elegans*, knockdown of *htz-1* by RNAi caused expression of genes dependent on the FoxA transcription factor PHA-4 to be delayed [23]. Furthermore, HTZ-1 and components of the *C. elegans* Swr1 complex (SSL-1) required for HTZ-1 deposition have been identified in genetic screens for suppressors of vulval induction, a process highly dependent on precise timing of transcriptional cascades and tightly coordinated with cell divisions [24],[25].

Another clue to the function of H2A.Z may lie in the fact that it is required for viability in all metazoans tested [26]–[29], but is not required for viability in single-celled yeast. A lack of H2A.Z during metazoan development typically causes defects that lead to late embryonic lethality [23],[26],[27],[29],[30]. This is consistent with expression of H2A.Z in mice, where the undifferentiated cells of the inner cell mass have low H2A.Z protein levels, with H2A.Z protein levels increasing as the cells differentiate into extraembryonic endoderm [17].

Whether H2A.Z has been associated with gene activation or repression in one study versus another may not represent a universal regulatory function for H2A.Z, but may instead be a reflection of the specific biological conditions under which the function of H2A.Z was observed in a given experiment, and the temporal resolution of the particular assays employed. In this light and with a focus on development, we used Chromatin ImmunoPrecipitation on DNA microarrays (ChIP-chip), genetic mutation, and RNAi to interrogate the function of HTZ-1 during embryogenesis in *C. elegans*.

## Results

### HTZ-1 Is Required for *C. Elegans* Development: As the Maternal Contribution of HTZ-1 Decreases, the Severity of Developmental Defects in Offspring Increases

**Figure 1:**
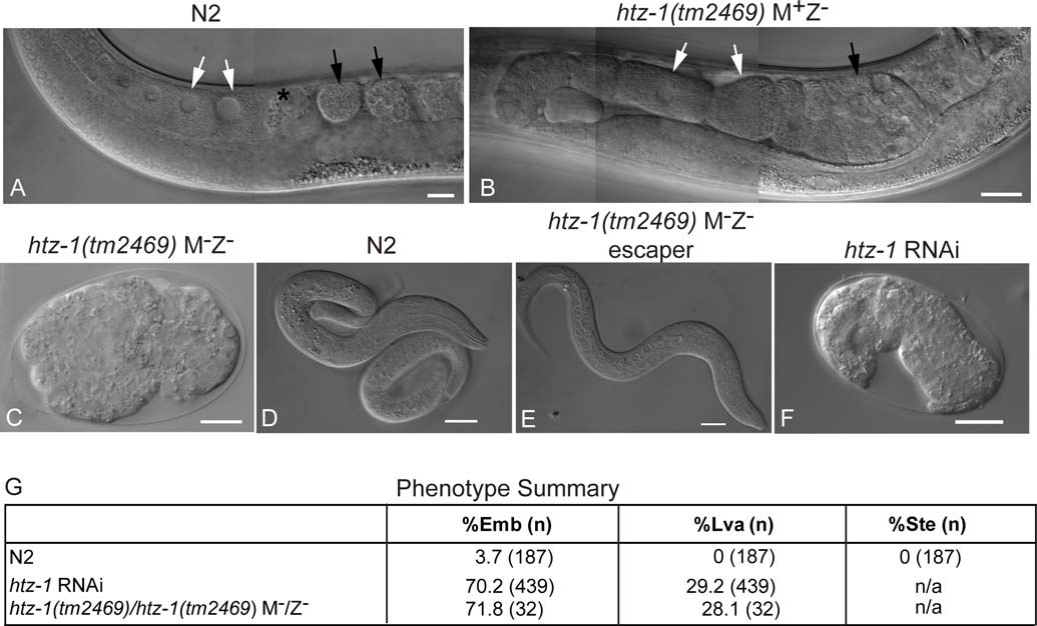
Maternal contribution of HTZ-1 is essential for development. Nomarski differential interference contrast (DIC) images of (A) N2 adult gonad, (B) *htz-1(tm2469)* M^+^Z^−^ adult gonad, (C) *htz-1(tm2469)* M^−^Z^−^ embryo (D) N2 embryo, (E) *htz-1(tm2469)* M^−^Z^−^ arrested L1 larva, (F) *htz-1* RNAi treated *eri-1(mg366)* embryos. White scale bars indicate 10 µm. (G) Summary of phenotypes for *htz-1(tm2469)* M^−^Z^−^ and *htz-1* RNAi. Emb, embryonic lethal; Lva, larval arrest; Let, lethal. Note that *htz-1(tm2469)* M^−^Z^−^ embryos remain *in utero*, but were excised for examination by microscopy. White arrows indicate oocytes, black arrows indicate fertilized embryos, and the asterisk indicates the spermatheca.

HTZ-1 knockdown by RNAi has been previously shown to cause embryonic lethality [23]. To further characterize the function of HTZ-1 (R08C7.3) in *C. elegans* development, we analyzed animals harboring a deletion in the *C. elegans htz-1* gene. The mutant *htz-1(tm2469)* contains a deletion of 345 bp of the *htz-1* gene, thereby eliminating 97 of the 140 predicted amino acids and making it a likely genetic null. The majority of homozygous *htz-1(tm2469)* offspring from *htz-1(tm2469)/+* heterozygotes (denoted as maternal +; zygotic −, or M^+^Z^−^) animals are rescued from embryonic lethality by a maternal contribution of HTZ-1. These rescued animals develop into worms exhibiting grossly normal morphology and germ cell proliferation until late adulthood (Figure 1A–B). Of the M^+^Z^−^ animals that reach adulthood, 80% are sterile and do not generate any embryos, instead producing unfertilized oocytes that eventually fill the uterus (Figure 1B). In 20% of the rescued animals, *M^−^Z^−^* embryos are observed in the uterus (Figure 1C). None of the embryos produced by *M^+^Z^−^* mothers were expelled from the uterus onto plates, indicating that the *M^+^Z^−^* mothers have an egg-laying defect (Egl). Somewhat unexpectedly, 28% of the M^−^Z^−^ embryos (n = 32) progressed through embryogenesis to produce a few hatched larvae. All of these M^−^Z^−^ escapers arrest at the first larval stage (Figure 1D–E). The M^−^Z^−^ embryos that hatched tended to arise from the first few eggs produced by *M^+^Z^−^* mothers, suggesting that in these animals HTZ-1 were still maternally provided at very low levels, but subsequent divisions of the germ cell precursors diluted HTZ-1 such that later embryos received a level below that required for viability.

**Figure 2:**
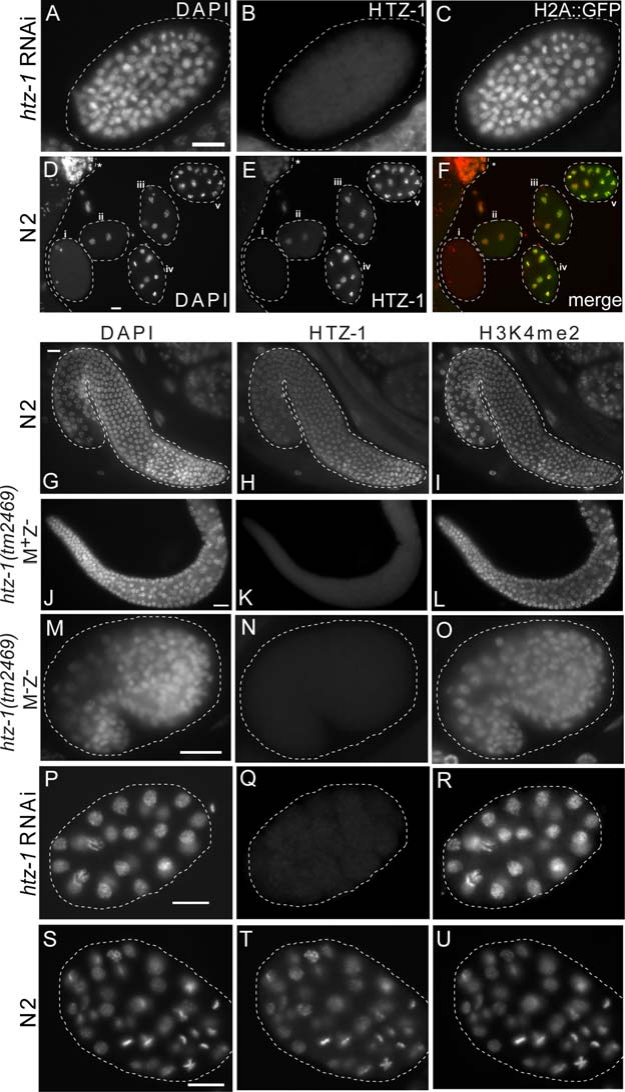
Immunofluorescence of *htz-1(tm2469) *and *htz-1* RNAi treated animals. H2A::GFP embryo treated with *htz-1* RNAi and stained with (A) DAPI, (B) anti-HTZ-1, and (C) anti-GFP. Immunofluorescence of early N2 embryo progression (D) DAPI, (E) anti-HTZ-1, and (F) merged DAPI (red) and HTZ-1 (green). In panels D-F: (i) fertilized egg, (ii) 2-cell embryo, (iii) 4-cell embryo, (iv) 8-cell embryo, (v) 16-cell embryo. Asterisk (*) indicates nearby germline cells. (G–U) Samples were stained with DAPI (column 1), anti-HTZ-1 (column 2) and anti-histone H3 dimethyl lysine 4 (column 3). In all images, anti-H3k4me2 serves as a positive control for antibody staining. (G–I) N2 gonad, (J–L) *htz-1(tm2469)* M^+^Z^−^ gonad, (M–O) *htz-1(tm2469)* M^−^Z^−^ embryo, (P–R) *htz-1* RNAi, and (S–U) N2 embryo. White scale bars indicate 10 µm.

The viability and semi-fertility of the *htz-1(tm2469) M^+^Z^−^* offspring suggested that the maternal load of HTZ-1 received by an embryo is sufficient to allow it to reach adulthood with defects limited to germ cells and specification of cells in post-embryonic lineages, for example vulval development. To test this, we targeted the maternal complement of *htz-1* mRNA using RNAi. Direct injection of dsRNA into the gonad of adult wild-type animals produced a more severe phenotype than was observed in M^+^Z^−^ offspring. Instead, the RNAi phenotypes are consistent with those observed in *htz-1(tm2469) M^−^Z^−^* embryos. Specifically, embryonic lethality was observed for 70% of the embryos, with the remaining animals dying as larvae (Figure 1F–G; Text S1). We verified that the *htz-1* dsRNA injections did not cross-react with H2A mRNA by showing that expression of a GFP-tagged version of H2A was not affected (Figure 2A–C). We interpret the progression of phenotypes resulting from either RNAi treatment or genetic mutation to indicate that HTZ-1 is required for both embryogenesis and for post-embryonic development. We propose that the occasional escape from lethality occurs due to perdurance of maternal HTZ-1 protein or RNA for as long as two generations, or in the case of RNAi, a failure to completely eliminate HTZ-1 protein or message in the offspring of injected mothers (Discussion).

### HTZ-1 Protein Is Present in All Cells and Increases in Abundance as Embryogenesis Progresses

HTZ-1 RNA is abundant in the form of a maternal contribution, and remains abundant throughout the majority of embryogenesis, suggesting that the function of HTZ-1 in development is widespread [31]. To investigate the distribution of HTZ-1 protein, we generated polyclonal antisera specific to a unique peptide sequence in the C-terminal region of HTZ-1 (Methods). The antibody recognized a single band of 15 kD on western blots of *C. elegans* protein extract, corresponding to the predicted molecular weight of HTZ-1 (Figure S1). Using these antibodies, we stained whole embryos and adults and found that HTZ-1 protein is present in all cell types throughout all stages of development. HTZ-1 protein levels are low in early embryos (1–12 cell), but increase as development progresses (Figure 2D–F).

HTZ-1 protein becomes detectably incorporated into chromosomes by the four-cell stage, coincident with the onset of zygotic transcription. This occurs in both wild-type and M^+^Z^−^ embryos, demonstrating that zygotic transcription of *htz-1* itself is not required for incorporation of HTZ-1 protein into chromatin. In wild-type adults, HTZ-1 protein is observed in both somatic and germline precursor cells (data not shown). No HTZ-1 protein was observed by immunofluorescence in M^+^Z^−^ adult gonads or their M^−^Z^−^ embryos (Figures G–O). In addition, no protein staining was observed in the offspring of animals injected with HTZ-1 RNAi (Figure 2P–R).

The low levels of HTZ-1 protein in young embryos, despite abundant *htz-1* mRNA, suggests that much of the maternal contribution is RNA-based, with HTZ-1 protein levels controlled post-transcriptionally (Figure 2D–F). Another case in which HTZ-1 protein levels do not depend on zygotic transcription can be inferred from the presence of HTZ-1 protein in the germline precursors (P lineage). In these cells, HTZ-1 protein is present in chromatin at levels comparable to the surrounding somatic blastomeres, despite the repression of zygotic mRNA production in the P lineage (Figure 2D–F) [32]. HTZ-1 protein is also observed in the chromatin of the primordial germ cells Z2 and Z3 (data not shown), which undergoes a dramatic erasure of histone H3 modifications during development [33],[34].

### HTZ-1 Incorporation Is Targeted to Predicted Transcription Start Sites

**Figure 3:**
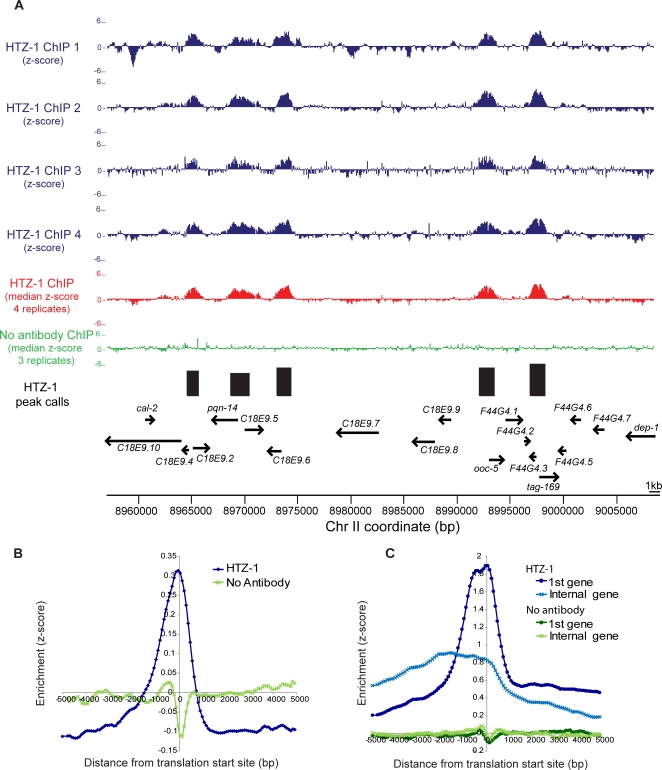
HTZ-1 localizes to sites of transcription initiation throughout the genome. (A). z-scores (calculated from the log_2_ ratio (ChIP/Input)) for each of the four HTZ-1 ChIPs (blue), the median HTZ-1 ChIP z-scores (red), and median no antibody ChIP (3 experiments; green) are plotted for 50 kb of chromosome II. Peaks of HTZ-1 binding are indicated by black bars, with gene annotations indicated below. Arrows specify the direction of transcription. (B) All genes were centered on the translation start site and HTZ-1 binding was averaged over all genes using a sliding window (window = 3 probes, step = 1 probe). A no antibody control ChIP is shown for comparison. The low-amplitude dip at the TSS in the no-antibody ChIP experiments is typical and has been reported previously [80] (C) Same as B, but for genes within operons.

To determine the genomic locations at which HTZ-1 functions, we performed ChIP-chip of HTZ-1 from extracts of wildtype N2 *C. elegans* embryos (Methods). For detection of ChIP-enriched loci, we used DNA microarrays consisting of 50-bp oligonucleotide probes that tile across the entire genome with 86-bp start-to-start spacing (Methods). Peaks of HTZ-1 binding were identified using ChIPOTle [35]. Throughout the genome, 5163 sites of HTZ-1 incorporation were found, with 85% of the peaks occurring within intergenic regions. Intergenic regions are defined as those that occur outside the boundaries defined by the translation start and stop sites of annotated transcripts or predicted genes. Under this definition, intergenic regions comprise 58% of the bases in the genome. Of the peaks within an intergenic region, 71% were within the 2-kb upstream of an annotated translation start site, 25% were within 2-kb of the translation stop, and only 4% were greater than 2-kb upstream of a translation start site. Among the 15% of peaks found to occur within an annotated transcription unit, most occurred near the 5′ end (median +545 bp downstream of the annotated translation start site). Therefore, like yeast Htz1, *C. elegans* HTZ-1 is preferentially incorporated into intergenic regions, specifically at promoters (Figure 3A).

We next investigated whether HTZ-1 was incorporated specifically at sites of transcriptional initiation. The majority of transcription initiation sites are not well-annotated in *C. elegans*, due in part to the prevalence of trans-splicing [36]. Therefore as a proxy for transcription initiation sites, we plotted HTZ-1 binding relative to annotated translation start codons. On average, the peak of HTZ-1 incorporation occurs just upstream of the translation start codon (Figure 3B), which we interpreted to indicate incorporation at or near sites of transcription initiation. To further test whether the observed signal represents sites of transcription initiation, we took advantage of a unique feature of the *C. elegans* genome. Approximately 15% of *C. elegans* genes are predicted to reside in operons that are transcribed as a large polycistronic pre-mRNA, which is then trans-spliced into mRNAs for the individual genes [37]. We plotted HTZ-1 incorporation relative to the first gene in operons, where transcription is expected to initiate, and also plotted incorporation relative to internal genes, where transcription is not expected to initiate. Indeed, HTZ-1 incorporation is generally observed upstream of the first gene in an operon, and does not generally occur upstream of internal genes (Figure 3C), indicating that *C. elegans* HTZ-1 is incorporated primarily at or near sites of transcription initiation. We also observed some important exceptions to this general rule, which are discussed below.

### Sites of HTZ-1 Incorporation Occur within at Least One-Third of Annotated Operons, Strongly Suggesting Independent Regulation of Internally Encoded Operon Genes

Currently most *C. elegans* operons are identified primarily by two criteria: the appearance of two or more genes in close proximity that are transcribed on the same strand, and the isolation of a downstream RNA transcript with an SL2 trans-spliced leader [38],[39]. In this way, a total of 1118 putative operons have been identified (genome release ws170). However, these criteria are imperfect, and do not provide information about genes that may be regulated both as part of an operon and by their own independent promoter. Independent transcription events within operons have been difficult to detect because the 5′ ends of mRNAs produced by either trans-splicing of a poly-cistronic mRNA or an independent transcription event are not readily distinguishable.

**Figure 4:**
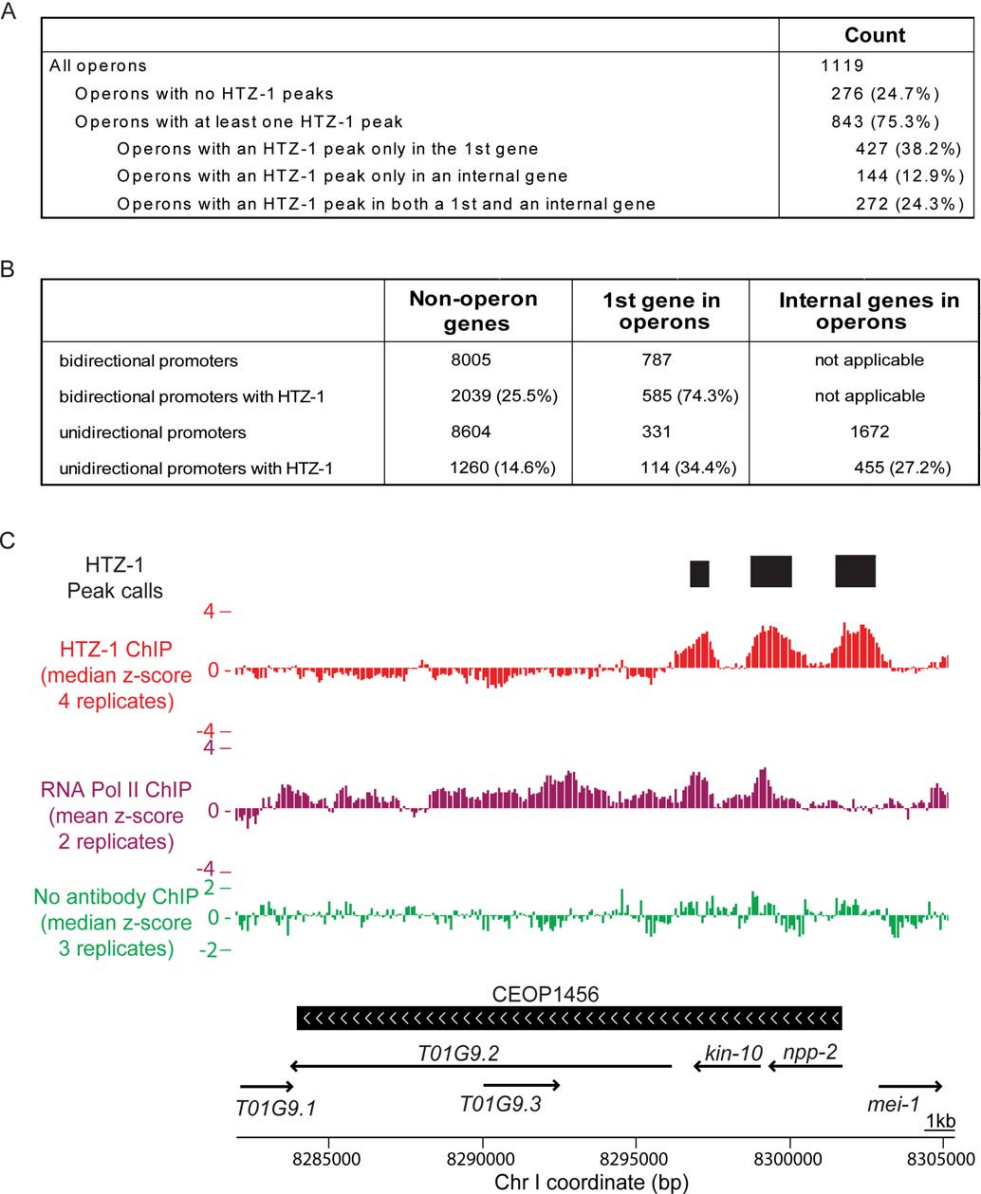
HTZ-1 incorporation predicts internal sites of transcription initiation in operons. (A) Table relating operons and sites of HTZ-1 incorporation. (B) Table relating genes within operons and sites of HTZ-1 incorporation. Bidirectional promoters are defined as those that are upstream of two divergently transcribed genes; unidirectional promoters are upstream of only one gene. (C) A genome browser view of the operon CEOP1456 (black bar with white arrows specifying the direction of transcription). Plotted are: median z-scores for the four HTZ-1 ChIPs (red), mean z-scores of two RNA Pol II replicates (purple), and median of three “no antibody” mock ChIPs (green). Peaks of HTZ-1 binding are denoted on the top track (black bars). Gene annotations indicated below (arrows).

To identify genes that are likely to be regulated both as part of an operon and individually, we examined incorporation of HTZ-1 at internally encoded genes of annotated operons. Overall, 75% of operons contained at least one HTZ-1 peak. A gene within an operon was more than twice as likely as a non-operon gene to have an HTZ-1 peak at its promoter (Figure 4A–B). Of operons containing at least one site of HTZ-1 incorporation, 85% contained a peak upstream of the first gene, as one might expect. However, 49% of operons with HTZ-1 incorporation at the first gene also exhibited an internal peak of HTZ-1 incorporation. This strongly suggests internal transcription start sites at 416 (37%) of the currently annotated operons (Figure 4B, Table S2). Because some operons contain multiple internal HTZ-1 peaks, this represents a total of 455 putative independently regulated genes within annotated operons. This is likely to be an underestimate, since the HTZ-1 localization data is derived only from embryonic extracts, meaning that genes and operons regulated specifically in adults or germ cells are not represented.

The unexpectedly high number of individually regulated genes within operons may to some extent reflect a mis-annotation of operons based on traditional criteria. To show that internal HTZ-1 incorporation can occur at verified operons, we examined CEOP1456, one of the first characterized operons, supported by cistronic RNA evidence [37],[40]. In this well-characterized operon, both HTZ-1 and RNA Polymerase II occupy the chromatin immediately upstream of the internal *kin-10* gene, strongly suggesting independent regulation (Figure 4C). Recently, differential regulation of genes driven by internal operon promoters was demonstrated using a GFP reporter assay [41]. We find that one-third of these internal promoters are occupied by HTZ-1 in embryos (Table S2). A time-course of the early embryonic transcription [31] provides evidence that genes within operons that contain multiple sites of HTZ-1 incorporation exhibit differential expression (Figure S2).

### During Embryogenesis, HTZ-1 Is Incorporated Upstream of 23% of *C. elegans* Genes, Which Function Preferentially in Growth and Development

In contrast to yeast, in which Htz1 is incorporated into nearly every promoter [42], our ChIP-chip data indicate that HTZ-1 is incorporated into the promoters of only 23% of *C. elegans* genes (Methods). To determine what might be held in common among the particular subset of genes that were occupied by HTZ-1, peaks were annotated to gene promoters, assigned Gene Ontology (GO) terms according to the nearest downstream gene, and evaluated with GO::TermFinder [43]. To avoid ambiguous gene assignments, only peaks annotated to unidirectional promoters or within coding regions were used in the input set. We found that GO terms associated with metazoan development and positive regulation of growth were strongly over-represented among HTZ-1 bound genes, while no overrepresented GO term was associated with the non-HTZ-1 bound genes (Table 1, Table S1). This finding suggests that HTZ-1 functions preferentially at the promoters of genes essential for growth and development.

**Table 1:** HTZ-1 is enriched at genes essential for growth and development.

GO Term	Associated Phenotypes	No. of HTZ-1 bound loci annotated to GO term (of 2986)	No. of all loci annotated to GO Term (of 22246)	Corrected p-value
Embryonic development ending in birth or egg hatching	Embryonic lethal (Emb), Maternal effect lethal (Mel)	750 (25%)	2650 (12%)	4×10^−102^
Positive regulation of growth rate	Developmental growth retarded (Gro)	387 (13%)	1300 (6%)	7×10^−54^
Larval development (sensu Nematoda)	Larval lethal (Lvl), Larval arrest (Lva)	435 (15%)	1615 (7%)	4×10^−48^
Gamete generation	Sterile progeny (Stp), Tumorous germline (Tum), Fewer germ cells (Fgc), Proximal germ cell Proliferation abnormal (Pro)	216 (7%)	681 (3%)	4×10^−33^
Cellular metabolic process		823 (28%)	4267 (19%)	2×10^−30^
Hermaphrodite genitalia development	Protruding vulva (Pvl), Everted vulva (Evl)	158 (5%)	502 (2%)	3×10^−23^
Sex differentiation	Male abnormal (Mab)	169 (6%)	564 (3%)	3×10^−22^

HTZ-1 peaks that could be unambiguously annotated to a single gene (either unidirectional promoters or coding regions) were selected. GoTermFinder [43] was used to find Gene Ontology (GO) Terms that are overrepresented among HTZ-1 associated loci. The number of HTZ-1 bound loci annotated to each GO term is shown in column 3, with the fraction of all HTZ-1 loci shown in parenthesis. For comparison, column 4 displays the total number of genes annotated to each GO term, with the fraction of all genes shown in parenthesis. The significance (corrected p-value) of overrepresentation is shown in column 5. All overrepresented GO terms with a corrected p-value less than 1×10^−20^ and farthest down the ontology tree are shown; for an expanded list see Table S2.

### HTZ-1 Occupancy at Promoters Is Linked to Transcriptional Activity and RNA Polymerase II Occupancy

**Figure 5:**
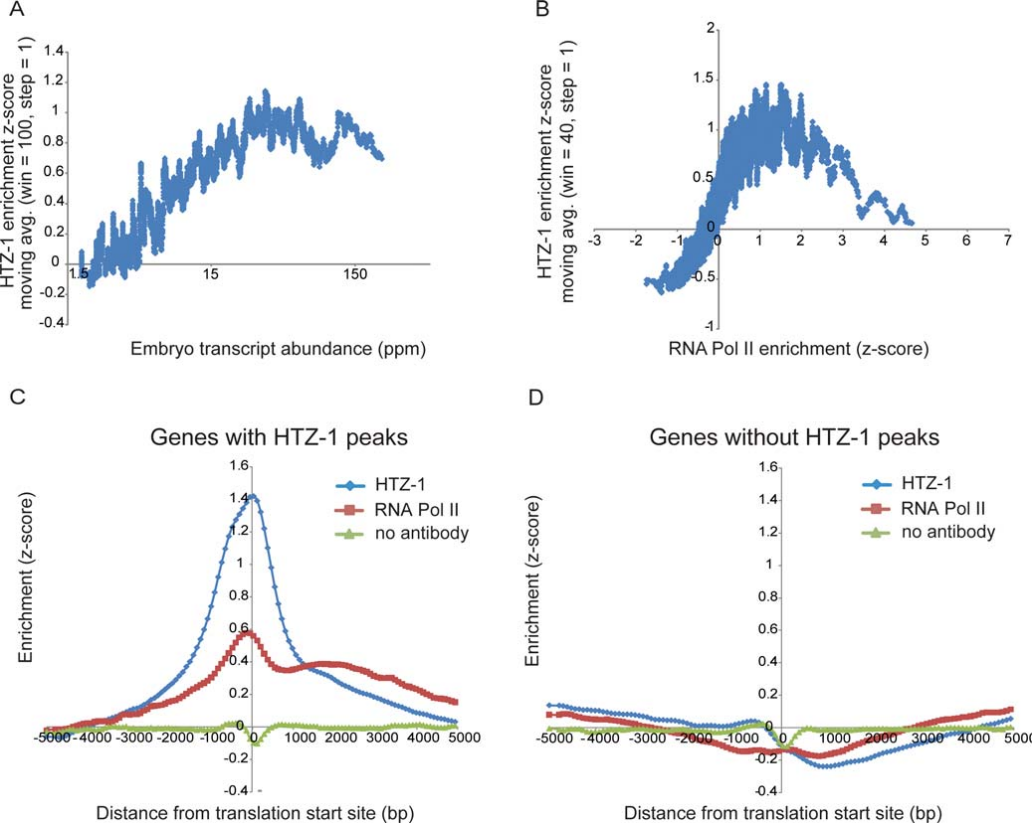
HTZ-1 occupancy correlates with transcript abundance and RNA Polymerase II occupancy. (A) Genes were sorted by transcript abundance (x-axis) and used to calculate a moving average of HTZ-1 ChIP enrichment z-scores (y-axis; window = 100, step = 1). HTZ-1 incorporation at each gene was scored by averaging all probes within 1 kb of the translation start site. The reported transcript abundance (ppm; parts per million) is the average of the last three time points from a published embryonic expression dataset [31]. (B) All genes were sorted according to the average RNA polymerase II promoter occupancy (z-scores, x-axis) used to calculate a moving average of HTZ-1 ChIP enrichment z-scores (y-axis; window = 40, step = 1). (C) The 4650 genes (which encompass 5567 translation starts nearby or within a peak) annotated to an HTZ-1 peak were centered on the translation start site. A sliding window average (window = 3 probes, step = 1 probe) was plotted for HTZ-1, RNA Polymerase II, and “no antibody” control ChIPs in the 10 kb surrounding the translation start. The low-amplitude dip at the TSS in the no-antibody ChIP experiments is typical and has been reported previously [80]. (D) The 15151 genes that were not annotated to HTZ-1 peaks were plotted as in (C). Note that the graphs in panels C and D represent the average RNA Polymerase II binding profile, but exceptions at individual genes exist (see Figure 6).

We next sought to examine the relationship between HTZ-1 occupancy at promoters and transcriptional activity during embryogenesis. We found that, in general, transcript levels [31] were positively correlated with HTZ-1 promoter occupancy (Figure 5A; Spearman rank-order correlation = 0.35). A positive correlation was also observed between RNA levels reported by a completely independent study [44] and HTZ-1 occupancy (Figure S3). Despite the positive overall correlation between occupancy and transcript levels, the relationship becomes negative at promoters of genes with very high transcript abundance (Figure 5A). This observation is consistent with a general loss of nucleosomes upstream of highly transcribed genes [45],[46].

We sought to establish a more direct link between HTZ-1 occupancy and transcription, so we determined the genome-wide occupancy of RNA polymerase II by ChIP-chip using an antibody specific to the C-terminal domain heptapeptide (8WG16, Methods). At gene promoters, HTZ-1 occupancy was strongly correlated with RNA Polymerase II occupancy (Figure 5B). In fact, the correlation was stronger than that observed between HTZ-1 occupancy and transcript levels (Spearman rank-order correlation = 0.57). Consistent with the correlation with transcript levels, at the promoters most highly occupied by RNA Polymerase II, the correlation with HTZ-1 occupancy was negative. Again, this observation is likely due to general nucleosome loss at the promoters of highly transcribed genes, for example those that encode the histone and ribosomal proteins [45],[46]. Temporal regulation gene expression during embryogenesis may also affect this correlation and is considered in the Discussion.

To further illustrate the relationship between HTZ-1 localization and polymerase occupancy, the 4650 genes with HTZ-1 incorporated into their promoters were aligned according to their translation start site, and average RNA polymerase II occupancy relative to the start site was plotted (Figure 5C). HTZ-1-occupied promoters were on average occupied by RNA Polymerase II, whereas genes lacking HTZ-1 were not (Figure 5D). At promoters occupied by HTZ-1, the average peak of HTZ-1 occupancy was at negative 12 bp relative to the translation start, while the average peak of RNA Polymerase II occupancy was slightly upstream at negative 98 bp (Discussion).

An important consideration in interpreting these relationships is that our experiments were performed using extract derived from a mixed population of embryos composed of many cell types. Therefore, our results are a projection of HTZ-1 occupancy throughout embryogenesis and represent a temporal and spatial average of the relationship between HTZ-1, RNA Polymerase II, and transcription (Discussion and Text S1).

### HTZ-1 Incorporation at Promoters Does Not Prescribe a Stereotypic Transcriptional Program

**Figure 6:**
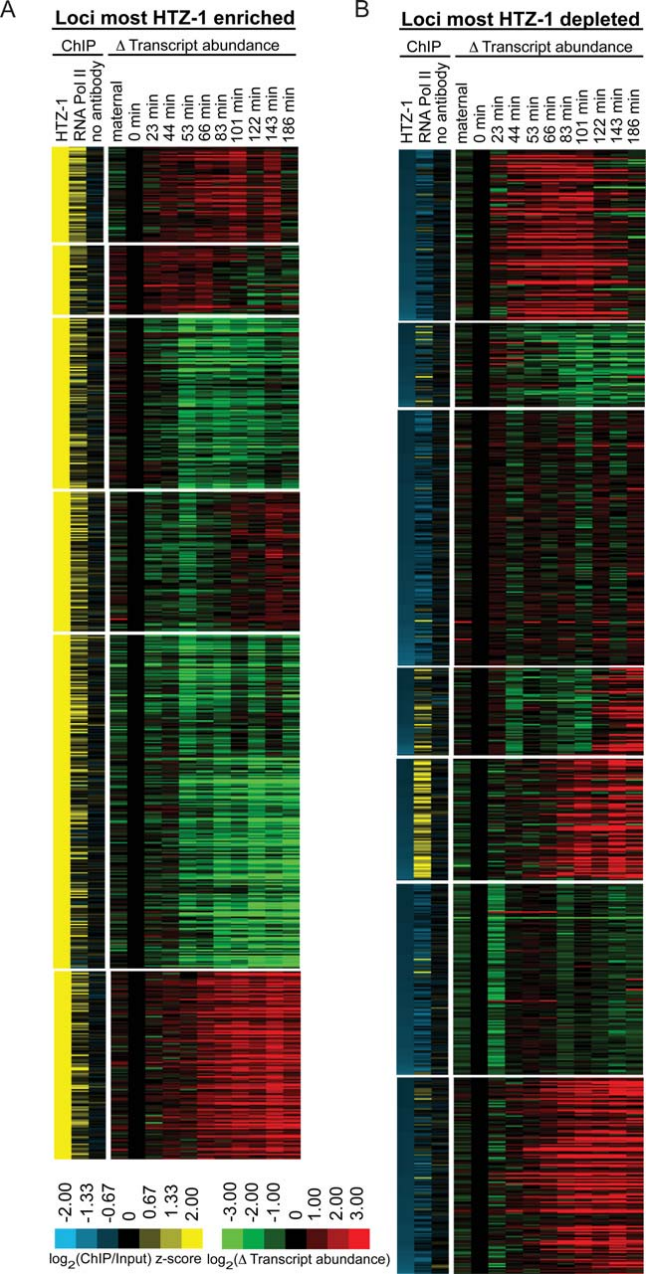
Genes downstream of HTZ-1 occupied promoters do not conform to a stereotypical expression pattern. (A) HTZ-1 occupancy at promoters was scored by averaging z-score ChIP values for all probes within 1 kb of the translation start site. Gene promoters with average z-scores greater than two were compared with a published expression dataset and are shown [31]. For each timepoint, the log_2_ of change in transcript abundance (timepoint/4-cell embryo) is shown. Genes with high maternal transcript abundance (absolute transcript abundance >100) were removed from analysis. K-means (k = 6) cluster analysis was performed with Cluster [78] using spearman rank correlation as a distance metric with 1000 iterations and visualized with Treeview [79]. (B) Same as A, but for promoters lacking HTZ-1 (z-score<−0.87).

To examine if HTZ-1 occupied promoters direct a stereotypic pattern of gene expression, we compared HTZ-1 occupancy, RNA Polymerase II occupancy, and transcription at each gene using a published time-course of transcript abundance during embryonic development [31]. Promoters occupied by HTZ-1 were clustered according to our RNA Polymerase II promoter occupancy data and the change in transcript abundance relative to the onset of zygotic transcription. To avoid ambiguity, transcripts that were highly maternally loaded (>100 parts per million (ppm)) were removed from analysis. Consistent with the aggregate analysis, RNA Polymerase II is abundant at most HTZ-1 occupied genes (Figure 6A), while promoters at which HTZ-1 is not incorporated generally lack RNA Polymerase II (Figure 6B). However, a large proportion of genes downstream of promoters occupied by both HTZ-1 and RNA polymerase II produce low transcript levels (Figure 6A), and conversely some genes produce high transcript levels despite low levels of HTZ-1 and RNA polymerase II at their promoters (Figure 6B; Discussion). Therefore, while HTZ-1 is strongly linked to RNA Polymerase II occupancy in aggregate, HTZ-1 bound promoters do not specify a stereotypic pattern of transcriptional regulation during development, suggesting that RNA polymerase occupancy and transcript levels are decoupled at some promoters.

### HTZ-1 Is Under-Incorporated on the X Chromosome

**Figure 7:**
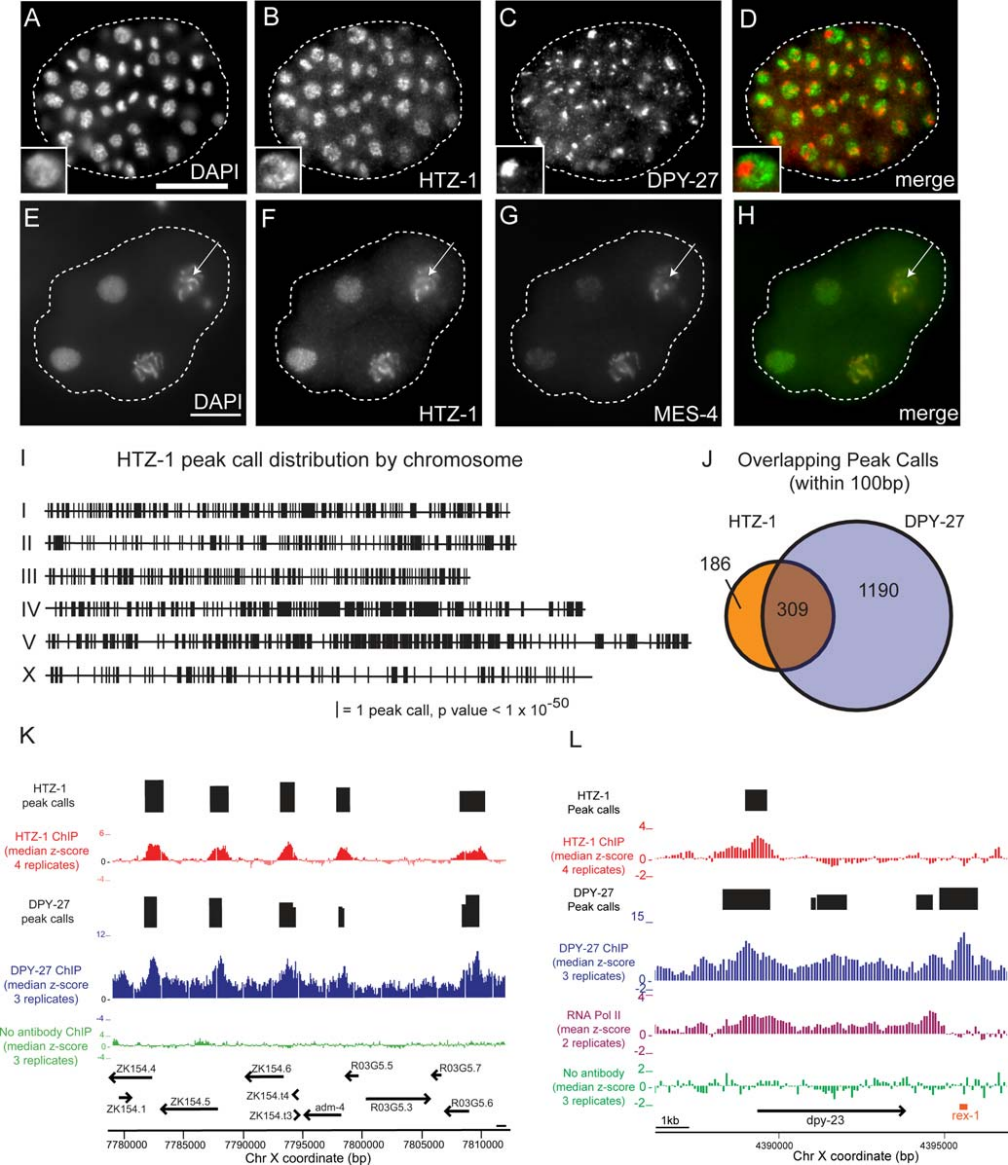
There are fewer sites of HTZ-1 incorporation on the X chromosome, but those that occur generally co-localize with the Dosage Compensation Complex. Immunofluorescence staining of an N2 embryo with (A) DAPI, (B) anti-HTZ-1, and (C) anti-DPY-27. (D) Merged image, HTZ-1 (green) and DPY-27 (red). One cell is enlarged 2× to highlight the distinction between DPY-27 and HTZ-1 staining (lower left). Immunofluorescence staining of a 4-cell N2 embryo with (E) DAPI, (F) anti-HTZ-1, and (G) anti-MES-4. (H) Merge of HTZ-1 (red) and MES-4 (green). Arrows point to the X chromosome. White scale bars indicate 10 µm. While some HTZ-1 incorporation on X is evident, note that the X is the brightest chromosome in the field by DAPI, but that HTZ-1 staining is brighter for other chromosomes, particularly the autosome at the bottom of the nucleus, than X. (I) Sites of HTZ-1 binding plotted along the chromosomes. Each line denotes one peak found with ChIPOTle using a Bonferroni corrected p-value of 1×10^−50^. (J) Locations of HTZ-1 incorporation were compared with DPY-27 peak distribution [51]. The Venn diagram displays the coincidence (peak maximum probes occurring within 100 bp) between the 495 HTZ-1 peaks and 1499 DPY-27 peaks on X. (K) A browser view of a 50-kb region of the X chromosome. Median z-scores (ChIP/Input) derived from four HTZ-1 ChIPs (red), three DPY-27 ChIPs (blue) [51] and three “no antibody” ChIPs (green) are plotted. HTZ-1 and DPY-27 binding peaks (black bars) and gene annotations (arrows) are indicated. (L) A browser view of the *dpy-23* locus; same as (K) with the addition of RNA Polymerase II ChIP (purple). The *rex-1* DCC recruitment element is shown in orange.

The sex chromosomes are often sites of specialized chromatin, harboring unique histone variants and chromatin modifications. To determine whether HTZ-1 was differentially localized to X, we co-stained embryos with anti-HTZ-1 in combination with either anti-DPY-27, which marks the X chromosomes in embryos of greater than about 30 cells (Figure 7A–D), or anti-MES-4, which marks the autosomes but not X chromosomes in early embryos (Figure 7E–H). In embryos that had initiated somatic dosage compensation, HTZ-1 incorporation was noticeably reduced on the X chromosomes, which was marked by DPY-27 staining (Figure 7A–D). However, co-staining with MES-4 revealed HTZ-1 under-representation on X even before the onset of somatic dosage compensation (Figure 7E–H). These results indicate that in both early-stage embryos before the onset of dosage compensation and late-stage *C. elegans* embryos after dosage compensation is established, there is significantly less HTZ-1 associated with the X than with autosomes.

We next aimed to ensure that reduction of HTZ-1 we observed on the X chromosome by immunofluorescence was not due to epitope exclusion. This concern was prompted by reports that mammalian H2A.Z on the inactive X chromosome is ubiquitylated, and that this modification can interfere with recognition by antibodies raised against a C-terminal peptide epitope [19]. Our antisera were also raised against a C-terminal peptide. To address this concern, we co-stained embryos expressing a HTZ-1:YFP transgene with anti-DPY-27 and anti-YFP antibodies. We observed a similarly reduced YFP signal coincident with regions of DPY-27 signal. This serves as independent evidence that within in the same nucleus, X chromatin has less HTZ-1 incorporation than autosomes (Figure S4).

### HTZ-1 Incorporation on X Is Largely Coincident with Binding of the Dosage Compensation Complex (DCC)

Two explanations for the under-incorporation of HTZ-1 on X immediately come to mind. One is that less HTZ-1 is incorporated on X as part of the *C. elegans* dosage compensation mechanism. A second explanation, which we favor for the reasons presented below, is that genes important for development, whose promoters tend to be occupied by HTZ-1, are under-represented on the X chromosome [47]–[50]. To distinguish these possibilities, we examined at high resolution the sites of HTZ-1 incorporation on the X chromosomes relative to the autosomes (Figure 7I).

One possible variation of the “dosage compensation” hypothesis predicts that sites of HTZ-1 incorporation are excluded or diminished on X as a consequence of the transcriptional repression imposed by the DCC. In this case, one would expect HTZ-1 occupancy on X to be excluded from sites occupied by the dosage compensation machinery [51]. Contrary to this prediction, we found strong co-localization of HTZ-1 incorporation and DCC binding, such that over 62% of HTZ-1 peaks are coincident with a DPY-27 peak (Figure 7J–K, Figure S7). The highly concordant binding pattern of HTZ-1 and the DCC on X would appear to rule out a function for HTZ-1 as a direct negative regulator of DCC binding to autosomes (Discussion).

We then considered the possibility that HTZ-1 incorporation is in fact a requirement for the loading of the DCC onto X. However, there are far more sites of DCC localization on X than HTZ-1 incorporation, meaning that most DCC-bound loci are not sites of HTZ-1 localization. For example, while both HTZ-1 and DPY-27 are incorporated at the X-linked *dpy-23* promoter, HTZ-1 is not incorporated at the well-characterized DCC recruitment site *rex-1*, which occurs just 5 kb downstream of *dpy-23* (Figure 7L) [51],[52]. We also examined in more detail *apl-1* and *lin-15*, two of the few genes known with some certainty to be dosage compensated [53],[54]. Although the DCC and RNA Polymerase II are present at both loci, HTZ-1 is present at *lin-15*, but not at *apl-1* (Figure S5), reinforcing the interpretation that HTZ-1 is not required for dosage compensation. Conversely, the under-representation of HTZ-1 on X is not dependent on the dosage compensation process, because it is evident in the germline and before the onset of somatic dosage compensation (Figure 7H).

### HTZ-1 Is Incorporated at Fewer Sites on X, but Individual Sites of HTZ-1 Incorporation on X Do Not Differ in Any Quantifiable Way from Sites of Incorporation on Autosomes

The alternative “developmental gene” hypothesis for the under-incorporation of HTZ-1 on X is based on the observation that only about half as many essential genes occur on X as would be expected to occur on an autosome of the same size (201 vs. 562 expected, wormbase release ws170) [47]–[50]. This hypothesis predicts that there would be fewer sites of HTZ-1 incorporation on X, but that those that do occur on X behave like those on autosomes. The X harbored 495 HTZ-1 peaks, about half the number expected from a hypothetical autosome with the size and gene density of X (p-value = 2.05×10^−43^ and 8.09×10^−93^ respectively, Figure 7I). There was no significant difference between the median height and width of HTZ-1 peaks on X (z-score = 2.28 and 774 bp, respectively) as compared to the median height and width of HTZ-1 peaks on autosomes (z-score = 2.20 and 860 bp, respectively) (Figure S8). This indicates that while HTZ-1 incorporation occurs at fewer loci on X, where it does occur the degree of incorporation is the same as the autosomes.

### HTZ-1 Likely Has an Indirect, but Not Direct, Function in Dosage Compensation

The most parsimonious explanation for the under-representation of HTZ-1 on the X is that the types of genes that require HTZ-1 for proper regulation are themselves under-represented on the X chromosome. Nonetheless, HTZ-1 is likely to have an indirect function in the dosage compensation process by affecting the regulation of genes required for dosage compensation. Strong HTZ-1 incorporation is observed at the promoters of *sdc-1*, *sdc-2*, *sdc-3*, *dpy-27*, *mix-1*, and *dpy-30*, all of which are required for dosage compensation. Although any number of complex scenarios involving a direct relationship between HTZ-1 and the canonical dosage compensation process remain possible, we interpret the under-representation of sites of HTZ-1 localization on X to be a simple consequence of the under-representation of germline and developmentally important genes on the X chromosome (Discussion).

## Discussion

Using a combination of genetic mutation, RNAi, microscopy, and ChIP-chip, we have characterized the function and genomic distribution of the histone variant HTZ-1 in *C. elegans*. Our study examines several unresolved issues surrounding H2A.Z function during development, including its relationship to the process of dosage compensation and the function of H2A.Z at genes essential for embryogenesis. In addition, our study reveals unexpected properties of *C. elegans* genome organization and regulation.

### The Developmental Function of H2A.Z

The *C. elegans* genome has been shaped by the developmental programs it must coordinately execute. The general requirement of H2A.Z for development in metazoans suggests a function for H2A.Z in establishing or maintaining a specialized chromatin state at developmentally regulated promoters [27]–[30],[55]. In this study, we have established that HTZ-1 is incorporated upstream of genes critical for development, and that maternally provided HTZ-1 is sufficient for *C. elegans* embryogenesis. We infer by the progressively deteriorating phenotype suffered by offspring lacking HTZ-1 that HTZ-1 is required for both embryogenesis and post-embryonic development.

The function of HTZ-1 in pharyngeal organogenesis may provide a model for the mechanism by which HTZ-1 is generally required for *C. elegans* development. The development of the pharynx relies on precise temporal regulation of transcription activation, mediated in part by PHA-4, a FoxA transcription factor [56],[57]. HTZ-1 depletion enhances defects in pharyngeal organogenesis associated with loss of PHA-4, and activation of PHA-4-dependent promoters is delayed in the absence of HTZ-1 [23]. This is reminiscent of the delay of yeast *GAL* gene activation in the absence of Htz1 [10], and indicates a conserved role for H2A.Z in facilitating timely gene expression.

### H2A.Z, Transcription, and Polymerase Pausing

Previous genome-wide studies in yeast and other organisms have reached differing conclusions regarding the relationship between H2A.Z, RNA Polymerase II, and transcription [11], [12], [15], [16], [20]–[22],[42],[58],[59]. Functional divergence between yeast Htz1 and metazoan homologs are a possible source of the discrepancy. Consistent with this, *C. elegans* HTZ-1 is only 61% identical to yeast Htz1, but 77% identical to Drosophila H2Avd, and 83% identical to mouse or human H2A.Z (Figure S6).

In *C. elegans*, we found that HTZ-1 is incorporated specifically at promoters, where its occupancy is strongly correlated with RNA polymerase II occupancy, and to a lesser degree with transcript levels (see Text S1). This suggests that RNA polymerase II is present at some HTZ-1 occupied promoters without being linked to a corresponding increase in transcripts. One possible explanation is pausing of RNA polymerase II near initiation sites. This phenomenon is common in human and Drosophila cells [15], [60]–[62] but has not yet been established to occur in *C. elegans*. The 8WG16 RNA polymerase II antibody we used is probably not the appropriate choice for making conclusions about RNA Pol II pausing, because the antibody recognizes primarily the unphosphorylated RNA Pol II CTD, but it is known to have some cross-reactivity with both CTD-Ser5P and CTD-Ser2P. RNA Polymerase II pausing would be more appropriately examined with an independent, non-C-terminal domain RNA Pol II antibody or a CTD-Ser5P specific antibody. Nonetheless, using the 8WG16 antibody, we observed a small number of genes (about 300, or ∼1.5%) with promoter-restricted RNA Polymerase II.

A recent genome-wide study of the Drosophila H2A.Z homolog at mononucleosome resolution reported that an H2A.Z-containing nucleosome was often positioned just downstream of a paused RNA polymerase II [15]. Although we did not observe any relationship, positive or negative, between HTZ-1 occupancy and this putative paused state, peak HTZ-1 occupancy occurs about 80 bp downstream of peak RNA Pol II occupancy at promoters (Figure 5C). Thus, the putative poised state may in some cases be facilitated by HTZ-1, and could contribute to the efficient and timely activation of developmental promoters. Indeed, our data does not formally exclude the possibility that H2A.Z functions to dampen transcription [63]. In Drosophila and mammalian cells, H2A.Z plays a role in gene silencing by participating in the assembly of heterochromatin [64],[65]. While another study of *C. elegans* HTZ-1 argues against a repressive role [23], and we observe high levels of expression from many genes that contain HTZ-1 at their promoters, we cannot exclude the possibility that transcription at these loci would be even higher in the absence of HTZ-1.

### Targeting HTZ-1 to Promoters

One key question for future studies concerns how H2A.Z is directed to developmental promoters. Sequence-specific transcription factor binding at promoters is likely an important driver of Swr1-mediated H2A.Z incorporation [23],[66]. At the human p21 promoter, sites of p53 binding are occupied by H2A.Z and p400 (a human Swr1 homolog) and this enrichment is dependent on p53 binding [13]. In C. elegans, association of HTZ-1 with pharyngeal promoters is dependent upon the presence of promoter PHA-4 motifs [23]. This requirement of PHA-4 for HTZ-1 association may be one specific example of the general mechanism underlying the specificity of HTZ-1 for developmental promoters.

Studies in yeast implicate histone tail acetylation as another important factor. Histone H4K16 acetylation is a prerequisite for Htz1 association near yeast telomeres [16],[67]. Yeast Htz1 recruitment is reduced in the absence of Bdf1, a bromodomain containing protein that binds acetylated histone tails, and GCN5, a histone acetyltransferase that acetylates Histone H3 tails [11]. The NuA4 histone acetyltransferase complex, which interacts with Bdf1 and is responsible for bulk H4 acetylation and acetylation of Htz1 itself, shares multiple non-catalytic components with the Swr1 complex [11], [68]–[70]. Nucleosome free regions (NFRs) at promoters may also play a role. Htz1 was deposited at sites flanking NFRs, which often harbor 22-nt motif that contained a Reb1 transcription factor binding site [20]. Insertion of this motif at an ectopic location was sufficient for NFR formation and flanking Htz1 incorporation.

### Probable Internal Transcription Start Sites in Operons

The incorporation of HTZ-1 at sites of transcription initiation suggests that HTZ-1 may be useful for identifying previously unannotated promoters. Our observation of HTZ-1 incorporation upstream of subsequent genes within operons implies the existence of independently regulated internal promoters in at least one-third of all currently annotated operons. Alternatively, operons may be less prevalent than the current genome annotation indicates. Indeed, a recent publication found evidence for functionally distinct internal promoters at 66 out of 238 (27%) downstream operon genes tested [71], a proportion of operons similar to which we found internal HTZ-1 incorporation. Additionally, transcript evidence from a published time-course during early development [31] provides evidence for independent regulation of some internal operon genes (Figure S2).

### A Function for HTZ-1 in Dosage Compensation?

Immunofluorescence and ChIP-chip experiments reveal a significant under-incorporation of HTZ-1 on the X chromosome relative to the autosomes. We explored three explanations for this under-representation: differential detection of the HTZ-1 protein specifically on the X; a function for HTZ-1 in dosage compensation; or an under-representation of developmentally important genes, which tend to be HTZ-1 targets, on X [47]–[50].

The first possibility is reasonable because mammalian H2A.Z can be ubiquitylated on its C-terminus, and this mark distinguishes H2A.Z incorporated on the heterochromatin and silent X chromosome [19]. The C-terminal residues are conserved in *C. elegans* HTZ-1, and include the antigen to which the antibody was raised. However, analysis of HTZ-1::YFP localization, in which detection would not be affected by modification state, indicates that under-representation on X is not due to epitope occlusion by ubiquitylation or any other cause (Figure S4).

The second possibility concerns *C. elegans* dosage compensation, during which the two hermaphrodite X chromosomes undergo chromosome-wide reduction in expression to match the output of the single male X chromosome [54]. While we observed a high degree of overlap between sites of HTZ-1 incorporation and sites of DPY-27 binding, the converse did not hold true. Many sites of DCC binding occur in areas of no HTZ-1 incorporation, including known recruitment sites such as the *rex-1* locus. This suggests that Dosage Compensation Complex binding does not require HTZ-1. We interpret the extensive co-localization on X to indicate independent functions of HTZ-1 and the DCC, both of which act upstream of genes active during embryogenesis. This interpretation is further supported by very few instances of overlap between HTZ-1 and DPY-27 at sites away from promoters. For example, only nine HTZ-1 peaks on X overlap with the 219 DPY-27 peaks found in the region downstream of genes. Of the nine overlaps, all may be explained as having promoter function: six occurred in regions near another gene promoter, and the remaining three were bound by RNA Polymerase II despite the absence of a gene annotation. Finally, HTZ-1 is under-incorporated on X both prior to and subsequent to the activation of somatic dosage compensation during embryogenesis.

The simplest explanation for the under-representation of HTZ-1 on X is that during embryogenesis HTZ-1 and the DCC both tend to bind at the transcription initiation sites of active genes important for development (Figure 7K). Genes essential for development are approximately 2-fold under-represented on the X [47],[48],[50], which is consistent with the approximately 2-fold under-incorporation of HTZ-1 on X. Although there are fewer sites of HTZ-1 incorporation on X, the individual sites of HTZ-1 incorporation on X do not differ in any quantifiable way from sites of incorporation on autosomes (Figure S8).

A ChIP-chip or ChIP-seq experiment revealing similar under-representation of HTZ-1 on the male X would provide further evidence against the direct involvement of HTZ-1 in dosage compensation. As stated in the results, HTZ-1 is likely to have an indirect function in dosage compensation and many other developmental processes by virtue of its incorporation at a wide spectrum of developmentally important genes. Nonetheless, our results do not completely rule out a direct positive or negative role for HTZ-1 in dosage compensation complex targeting or function. For example, the HTZ-1 on X could be post-translationally modified differently from HTZ-1 on autosomes [19],[72],[73], thereby conferring a distinct function for HTZ-1 depending on where in the genome it is incorporated.

### Integrating Biochemical, Genetic, and Genomic Data Regarding H2A.Z

One challenge in understanding H2A.Z function is integrating very diverse types of data, each of which lends clues to H2A.Z function but has its own limitations. For example, our experiments were performed in an unsynchronized population of embryos composed of multiple cell types. Therefore, the results presented here are a static projection of the dynamic activation and repression events that are occurring at gene promoters. How this might be manifested in our dataset can be illustrated by considering a previous time-course study of HTZ-1 at the *myo-2* promoter [23]. HTZ-1 was not incorporated into the *myo-2* promoter when it was repressed, but was transiently incorporated at the onset of transcription. HTZ-1 was then lost as *myo-2* became highly expressed later in development. In a temporal projection of these results, as occurs in our dataset, it appears as if HTZ-1 was very weakly incorporated (if at all) into the *myo-2* promoter (Text S1). Our genome-wide data indicates a preferential incorporation of HTZ-1 at developmentally dynamic promoters, and a loss of HTZ-1 at very highly transcribed genes. We infer that the general conclusions implied by the previous time-course study conducted at the *myo-2* locus are now extended to the entire genome by our data [23].

The biophysical properties of nucleosomes containing H2A.Z provide clues about how H2A.Z could facilitate precise, coordinated developmental transcriptional programs. Incorporation of H2A.Z into an otherwise canonical nucleosome appears to have slight stabilizing effects, but incorporation of H2A.Z into nucleosomes containing H3.3, a mark of active transcription, is reported to cause instability [4],[5]. Furthermore, H2A.Z incorporation may alter associations between the histone proteins within the nucleosome, regulating the formation of higher-order chromatin fibers [18],[74]. The H3.3 mediated instability could allow H2A.Z to facilitate or maintain a nucleosome-free region at promoters upon activation, while the effect of H2A.Z on higher-order structures could promote the maintenance of transcription by promoting more precise nucleosome positioning at promoters or by promoting the assembly of a specialized higher-order chromatin state [4],[15],[18],[21],[42],[75]. In this way, HTZ-1 may aid the *C. elegans* embryonic genome in executing rapid transitions between quiescence and activity as developmental programs are executed.

## Materials and Methods

### Antibodies

The RNA polymerase II monoclonal antibody 8WG16 was obtained from Covance. A mouse ascites polyclonal anti-HTZ-1 antibody was made to the HTZ-1 specific C-terminal peptide (N-PGKPGAPGQGPQ-C) by Invitrogen-Zymed. Rabbit DPY-27 polyclonal antibodies used for immunofluorescence were generously provided by Dr. B.J. Meyer (UC Berkeley). Rabbit polyclonal MES-4 antibodies were generously provided by Dr. S. Strome (UC Santa Cruz). AlexaFluor donkey anti-mouse 488 IgG (Invitrogen A-21202), AlexaFluor donkey anti-rabbit 594 IgG (Invitrogen A-21207) were used as secondary antibodies for immunofluorescence. Anti-mouse HRP and ECL Plus (Amersham) were used for western blot visualization.

### Strains

ChIP-chip analysis was performed in the N2 Bristol strain. *htz-1(tm2469)* was obtained from Shohei Mitani and balanced over nT1(qIs51) IV,V. KW1665 (*htz-1(tm2469)* IV/*nT1(qIs51)* IV,V) is maintained by selecting GFP-positive heterozygotes. All strains were cultured under standard conditions [76] at 20°C using *E. coli* strain OP50 or HB101 as a food source.

### Nomarski DIC and Immunofluorescence Microscopy

Adult hermaphrodites gravid with embryos were dissected in 1× PBS (137 mM NaCl, 2.7 mM KCl, 8 mM Na_2_HPO_4_ and 2 mM KH_2_PO_4_) on a slide. Paraformaldehyde was then added to 5%. The slide was incubated at room temperature for 2 minutes with a cover slip in place, and placed on dry ice for approximately 20 min. The cover slip was removed rapidly with a razor, and the slide was then placed into 95% ethanol for 2 minutes, followed by incubation in PBST (1× PBS+0.1% Tween-20) for 30 minutes. Slides were incubated with primary antibody at 1∶500 (HTZ-1) or 1∶100 (DPY-27 and MES-4) dilution overnight and with secondary antibody (1∶500) for approximately 3 hours. 4′,6-diamidino-2-phenylindole (DAPI) was used to stain DNA. Slides were mounted using ProLong® Gold antifade reagent (Invitrogen P36934). Staining was visualized using a Leica DMRXA microscope outfitted with a Cooke Sensicam. Capture and analysis of immunofluorescence images was performed using either Volume Scan (Vaytek) and Image-Pro Plus (Media Cybernetics) or SimplePCI.2 (Hamamatsu Corporation) imaging software. Nomarski DIC microscopy imaging was performed with a Leica DMRXA microscope and SimplePCI.2 software.

### RNAi

dsRNA was generated by *in vitro* transcription reaction using a Promega RiboMAX Large Scale RNA Production Systems T7 kit (Promega #P1300). Direct injection of concentrated dsRNA into adult gonads was required to obtain significant levels of embryonic lethality and larval arrest, as standard feeding and soaking methods did not result in sufficient depletion of the maternal HTZ-1. 1.2 µg/µL *htz-1* dsRNA was injected into young adult *eri-1(mg366)* or N2 animals. The injected animals were allowed to recover and lay embryos overnight, then transferred to a new plate for collection and phenotypic scoring of affected embryos for 9–12 hrs. Following the 9–12 hour period, the adults and the embryos *in utero* were dissected and processed for immunofluorescence. The phenotypes of hatched larvae were observed and analyzed by DIC light microscopy 2–3 days after hatching. N2 animals were used for phenotype counts and staining experiments; *eri-1(mg366)* animals were used for DIC RNAi phenotype experiments.

### Chromatin ImmunoPrecipitation

Embryos were prepared by bleaching from gravid N2 adults grown in S-basal media liquid culture. Live embryos were cross-linked using 2% formaldehyde for 30 minutes at room temperature followed by quenching with 125 mM glycine for 15 minutes. Embryos were then washed twice with M9 Buffer, once by ChIP buffer, and frozen at −80°C. Extracts were prepared by resuspending embryo pellets in 1 volume ChIP Buffer (50 mM HEPES-KOH pH 7.5, 300 mM NaCl, 1 mM EDTA pH 8.0, 1% TritonX-100, 0.1% sodium deoxycholate, 10% glycerol, protease inhibitors (Calbiochem)), followed by dounce homogenization (50×) and sonication (4×, 1 s on, 0.5 s off, at 20% amplitude on ice) using a Branson Digital Sonifier 450. In a volume of 500 µL, 2 mg extract was used for each ChIP. 100 mg (5%) of the extract was set aside as “Input” and 400 µL elution buffer (0.1 M NaHCO_3_, 1% SDS) was added. Two (anti-RNA Pol II) or six (anti-HTZ-1) µg of antibody was added to each IP sample and incubated overnight at 4°C. Immune complexes were purified with 10 µL protein-A sepharose (Amersham) and washed 5 minutes with 1.5 mL of each of the following solutions: ChIP Buffer, ChIP Buffer with 500 mM NaCl, ChIP Buffer with 1 M NaCl (HTZ-1 IPs only), LiCl solution (10 mM Tris-HCl pH 8.0, 250 mM LiCl, 0.5% NP-40, 0.5% sodium deoxycholate, 1 mM EDTA), and TE (10 mM Tris-HCl pH 8.0, 1 mM EDTA). Samples were treated with 20 µg RNAse for 30 minutes at 37°C. IP samples were eluted twice with 200 µL elution buffer. NaCl was added to 200 mM and crosslinks were reversed by incubation overnight at 65°C. DNA was purified using Zymo DNA purification columns and amplified using LM-PCR [51].

### Microarrays and Data Extraction

Microarrays used were previously described (GEO GPL4614 and GPL4619; [51]). Four independent HTZ-1 ChIP biological replicates were performed, one of which was a dye-swap (ChIP 4). RNA Polymerase II ChIPs were performed from extracts used for HTZ-1 ChIPs 1 and 2. DPY-27 and “no antibody” datasets were published previously (GEO GSE6739; [51]). HTZ-1, RNA Polymerase II, and no antibody raw intensities were normalized by median centering log_2_ ratios (IP/input). Normalized log_2_ ratios from each experiment were converted to standardized z-scores and combined by taking the median of experiments. Raw data for HTZ-1 and RNA Polymerase II can be found at NCBI GEO accession number GSE10201. Peaks were derived using a Perl implementation of ChIPOTle (https://sourceforge.net/projects/chipotle-perl/) [35] using a window size of 500 bp, step size 86 bp, at a Bonferroni corrected p-value of 1×10^−9^. Any HTZ-1 peaks overlapping a peak found in the mock “No antibody” IP were removed from analysis. Peaks were annotated using Wormbase genome release 120 (Table S3). Maximum probe centers of peaks were annotated either to an intergenic or coding region, exclusively. Annotation distribution statistics were calculated using an unpublished *C. elegans* implementation of Cis-Element Annotation Software (CEAS) ([77], X. Shirley Liu, unpublished). Genome browser views were generated using the UCSC genome browser (http://genome.ucsc.edu), using the ce2 (March 2004)/ws120 genome build. Analysis for overrepresentation of Gene Ontology terms was done with GOTermfinder [43], accessed November 11, 2007 at http://go.princeton.edu/.

The figure of 23% of *C. elegans* genes being incorporated with HTZ-1 is based on annotating HTZ-1 peaks to the closest promoters and coding regions of 4650 genes (23.4%). In the case of bidirectional promoters (1800), both genes downstream were counted. Therefore, this number may be an overestimate if HTZ-1 functions only at one of the two genes in these cases.

### Transcription Analysis and Clustering

HTZ-1 or RNA Polymerase II occupancy at each gene promoter was scored by averaging all probes within a 1-kb window centered on translation start sites. Transcript abundance data was obtained from a published study [31] and compared by averaging the last 3 timepoints from this study. To avoid ambiguity from maternally loaded RNAs, genes with high maternal transcript abundance (>100 parts per million) were removed from the clustering analysis. Each time point was divided by the 0 minute time point (the onset of zygotic transcription) and log_2_ transformed. K-means clustering (k = 6, 1000 iterations, similarity metric = spearman rank correlation) was performed using Cluster 3.0 [78] and visualized using Treeview [79].

## Supporting Information

Figure S1 Anti-HTZ-1 antibody recognizes a single ∼15 kD protein.
(2.24 MB TIF)

Figure S2 Internal promoters identified by HTZ-1 occupancy are differentially expressed during early embryogenesis.
(1.54 MB TIF)

Figure S3 HTZ-1 occupancy at promoters is positively correlated with embryo expression.
(0.99 MB TIF)

Figure S4 Anti-HTZ-1 C-terminal antibody is not specifically excluded from the X chromosome.
(6.67 MB TIF)

Figure S5 HTZ-1 occupancy at the promoters of known dosage compensated genes apl-1 and lin-15A/B.
(1.91 MB TIF)

Figure S6 *C. elegans* HTZ-1 is more similar to *Drosophila* H2Avd and vertebrate H2A.Z than yeast Htz1.
(1.23 MB TIF)

Figure S7 HTZ-1 peaks are coincident with DPY-27 peaks on the X chromosome.
(1.26 MB TIF)

Figure S8 A comparison of mean HTZ-1 peak height and width between X and the autosomes.
(1.48 MB TIF)

Table S1 Complete list of all over-represented Gene Ontology Terms.
(0.09 MB PDF)

Table S2 The relationship between operons, operon genes, and HTZ-1 occupancy at promoters.
(0.10 MB PDF)

Table S3 HTZ-1 peak calls.
(0.76 MB XLS)

Text S1 Supplemental text.
(0.12 MB PDF)
