# Supplementary figures and images for: The Genomic Distribution and Function of Histone Variant HTZ-1 during C. elegans Embryogenesis
Source: PLoS Genet. 2008 Sep 12;4(9):e1000187. doi: 10.1371/journal.pgen.1000187 (PMC2522285; doi:10.1371/journal.pgen.1000187)

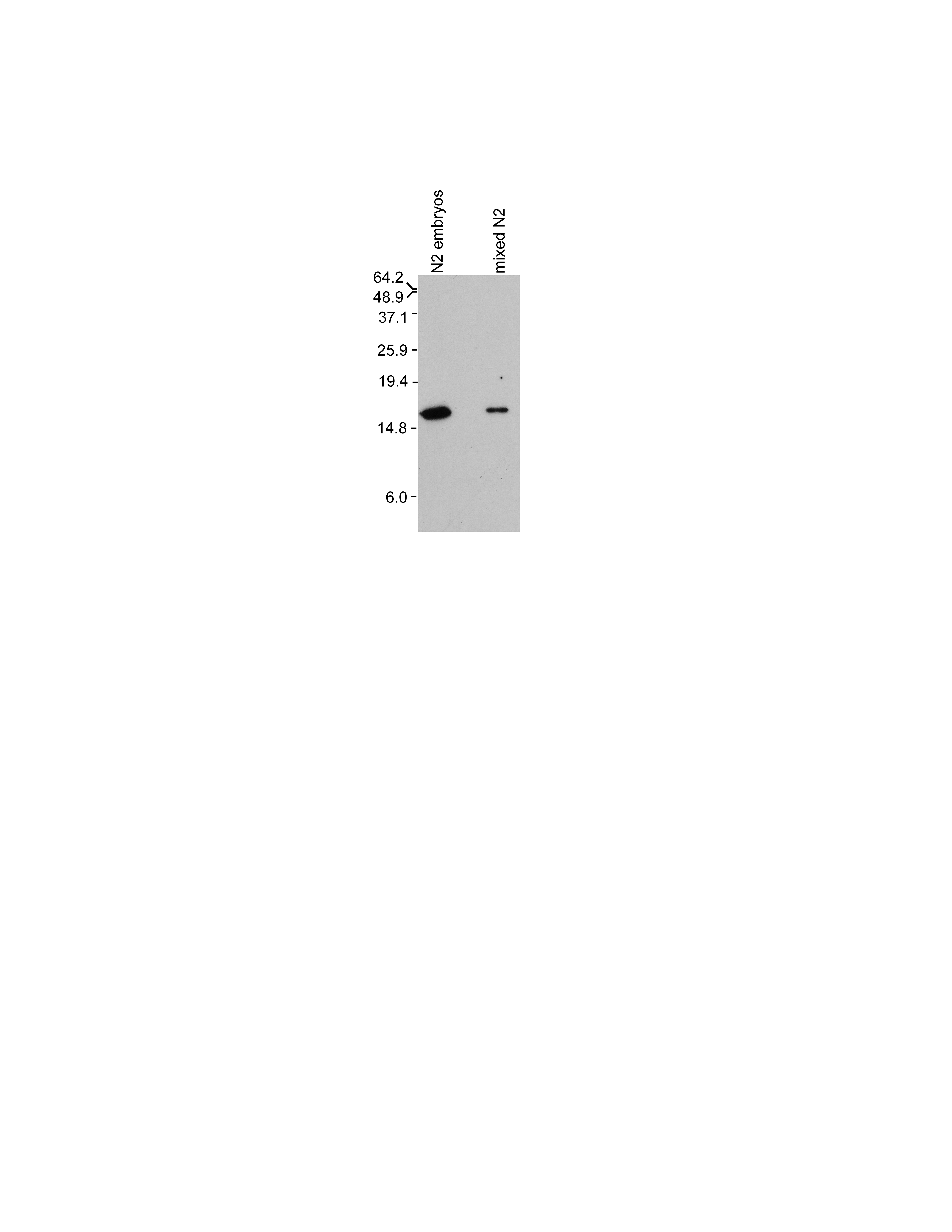

Supplement: Figure S1 — Anti-HTZ-1 antibody recognizes a single ∼15 kD protein. (2.24 MB TIF) [file pgen.1000187.s001.tif]

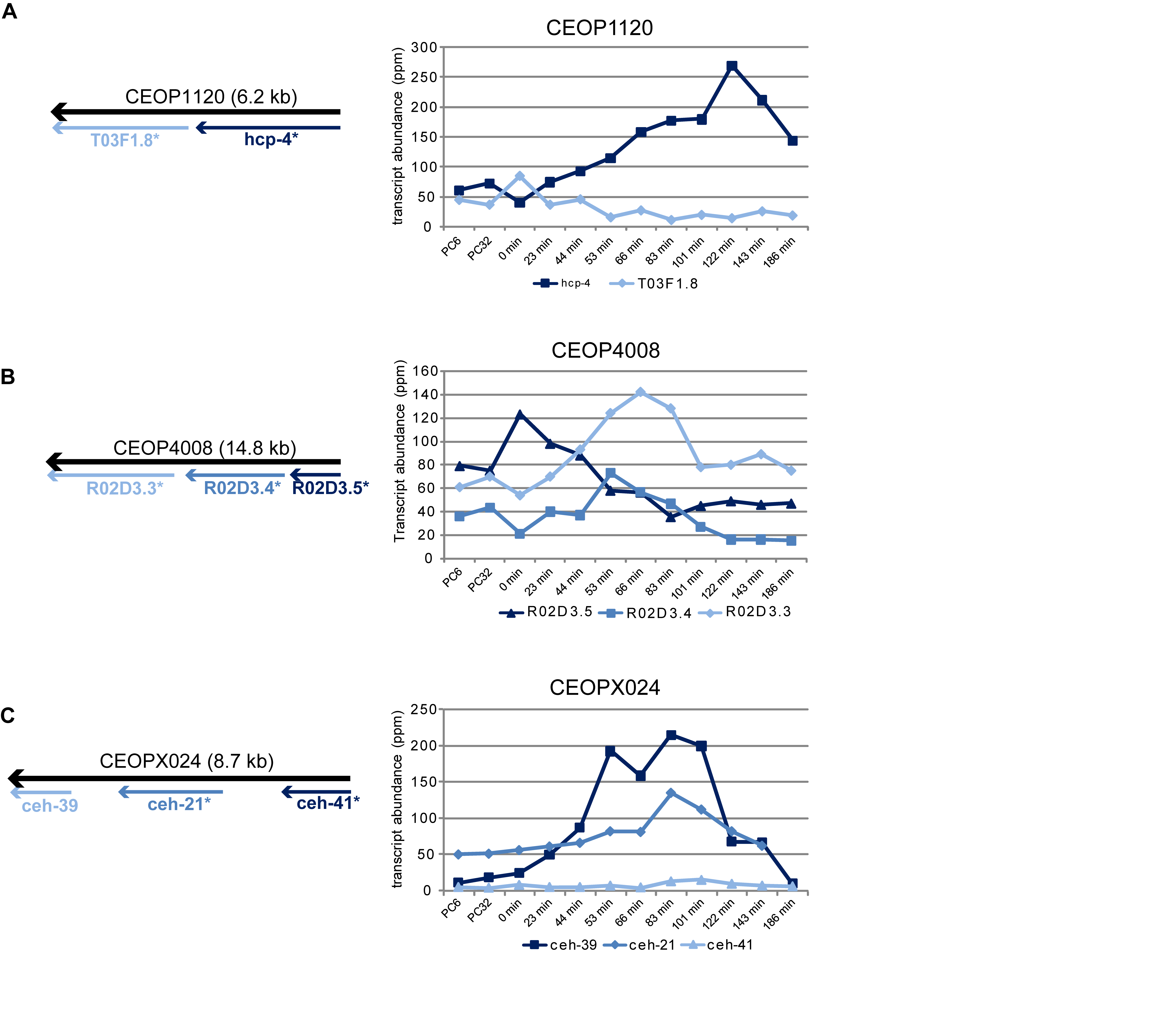

Supplement: Figure S2 — Internal promoters identified by HTZ-1 occupancy are differentially expressed during early embryogenesis. (1.54 MB TIF) [file pgen.1000187.s002.tif]

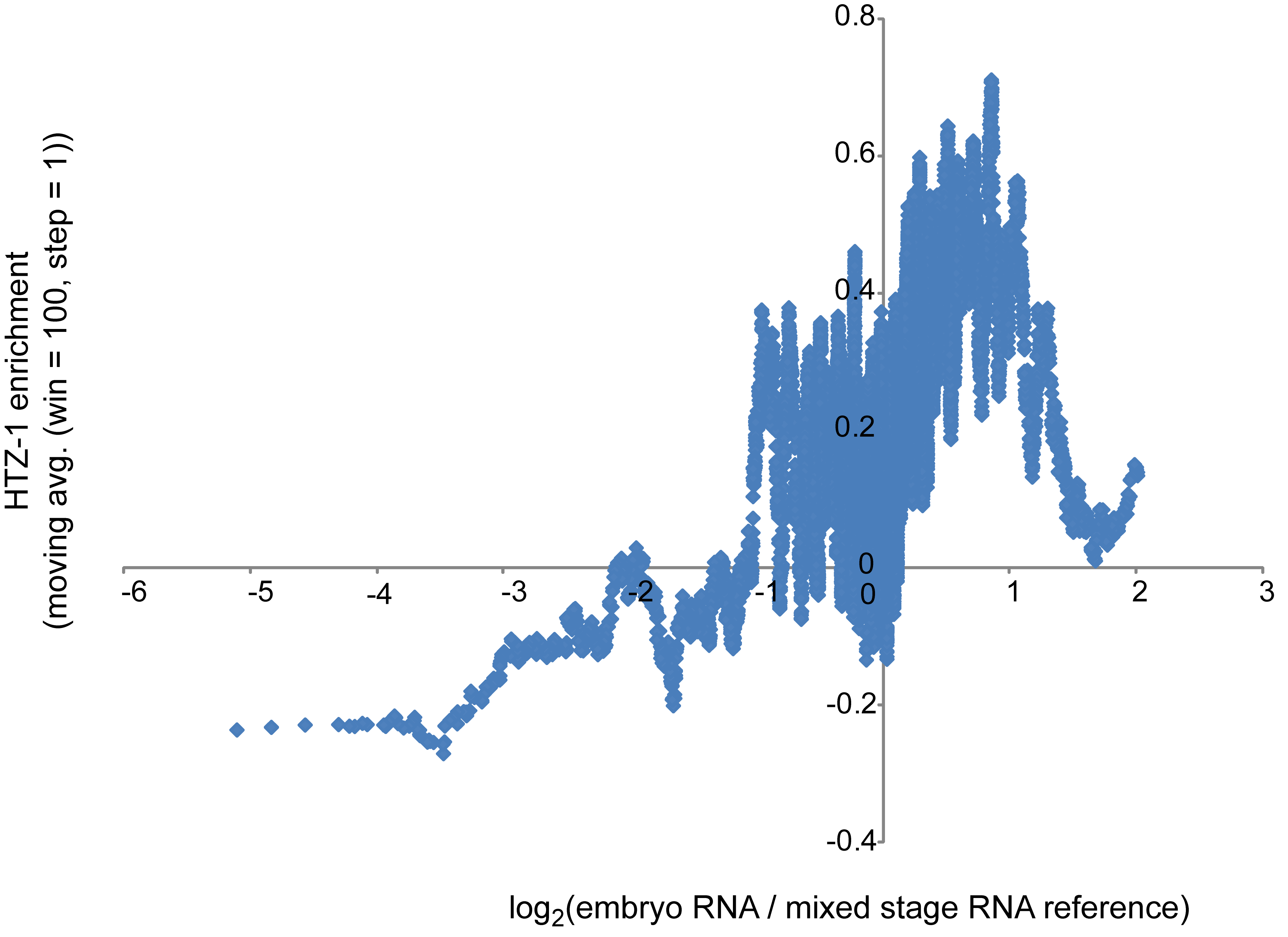

Supplement: Figure S3 — HTZ-1 occupancy at promoters is positively correlated with embryo expression. (0.99 MB TIF) [file pgen.1000187.s003.tif]

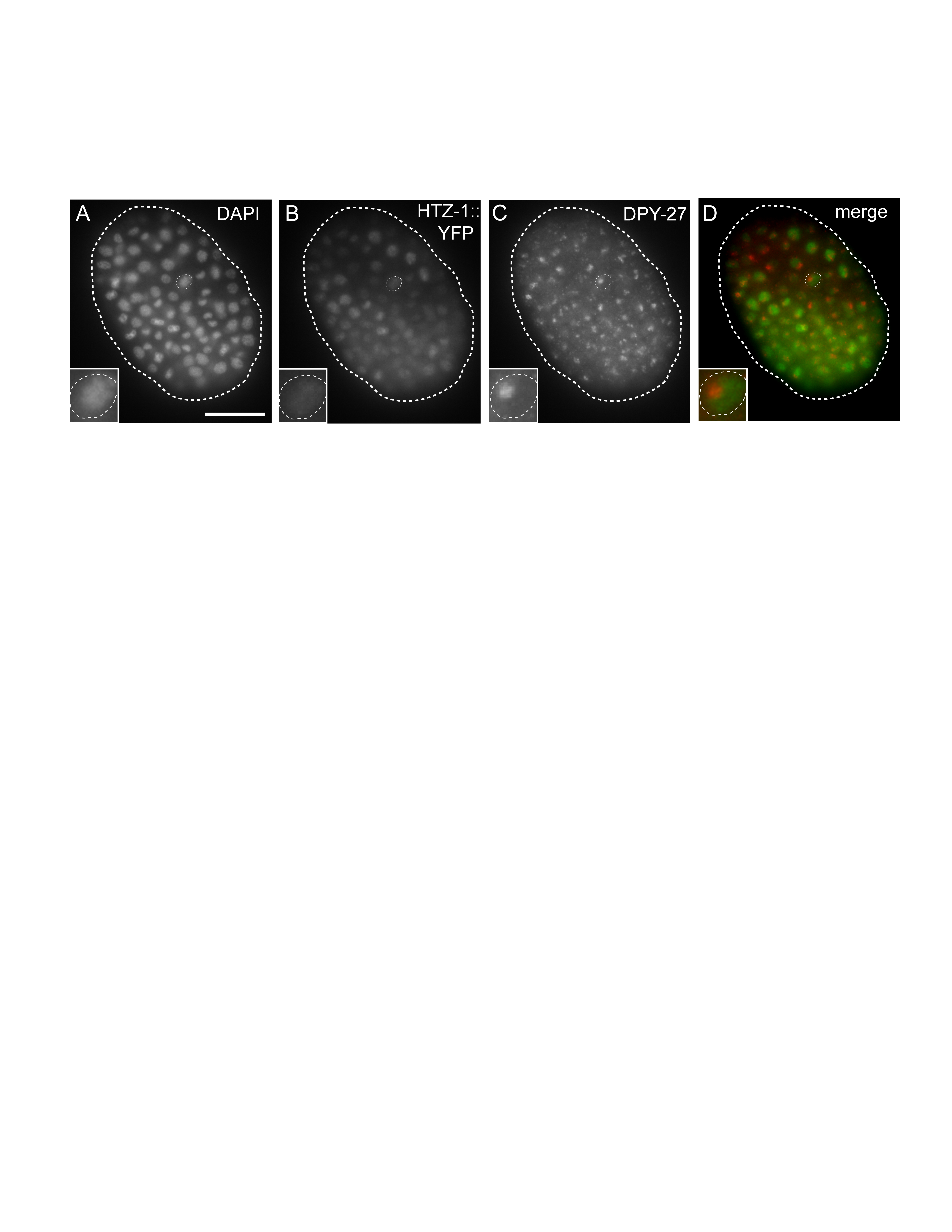

Supplement: Figure S4 — Anti-HTZ-1 C-terminal antibody is not specifically excluded from the X chromosome. (6.67 MB TIF) [file pgen.1000187.s004.tif]

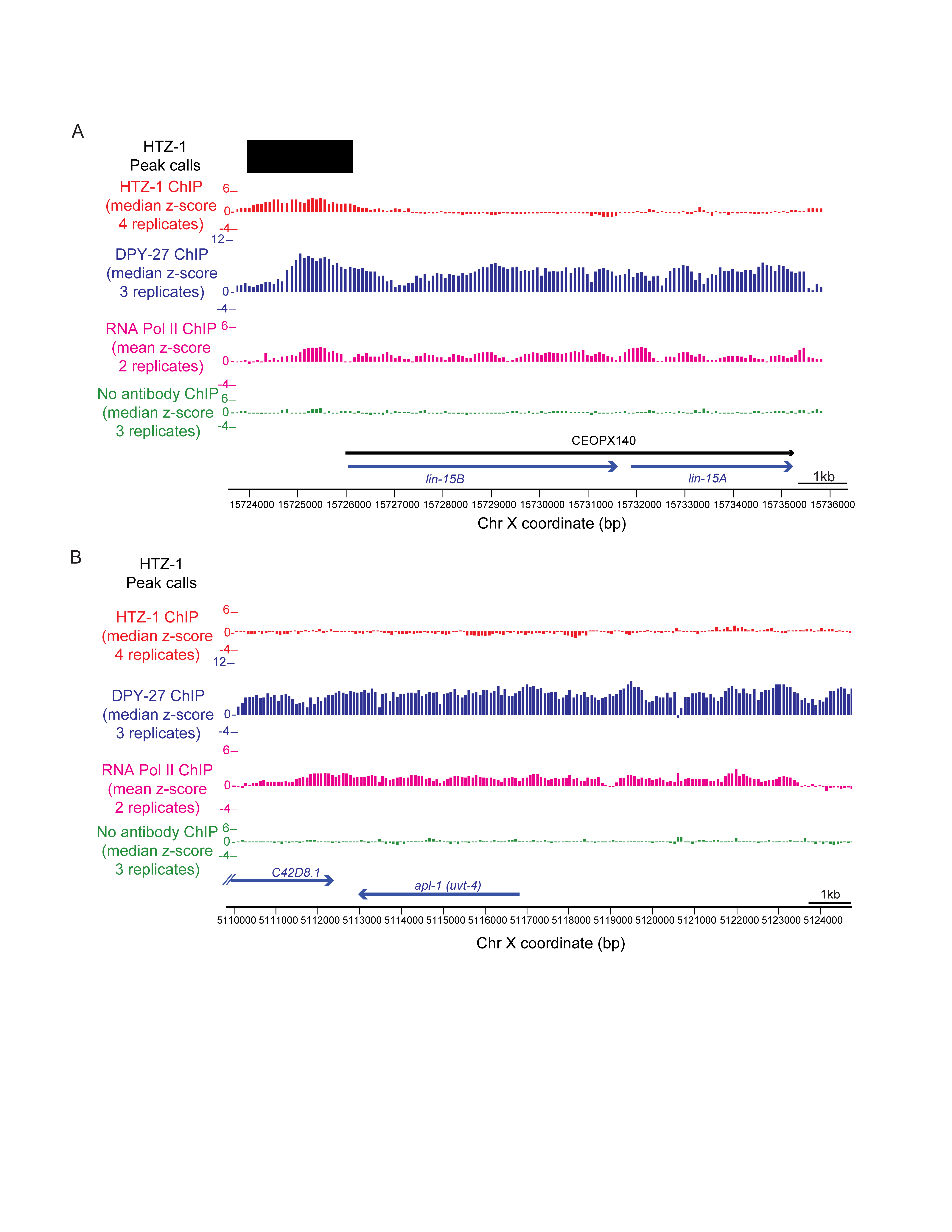

Supplement: Figure S5 — HTZ-1 occupancy at the promoters of known dosage compensated genes apl-1 and lin-15A/B. (1.91 MB TIF) [file pgen.1000187.s005.tif]

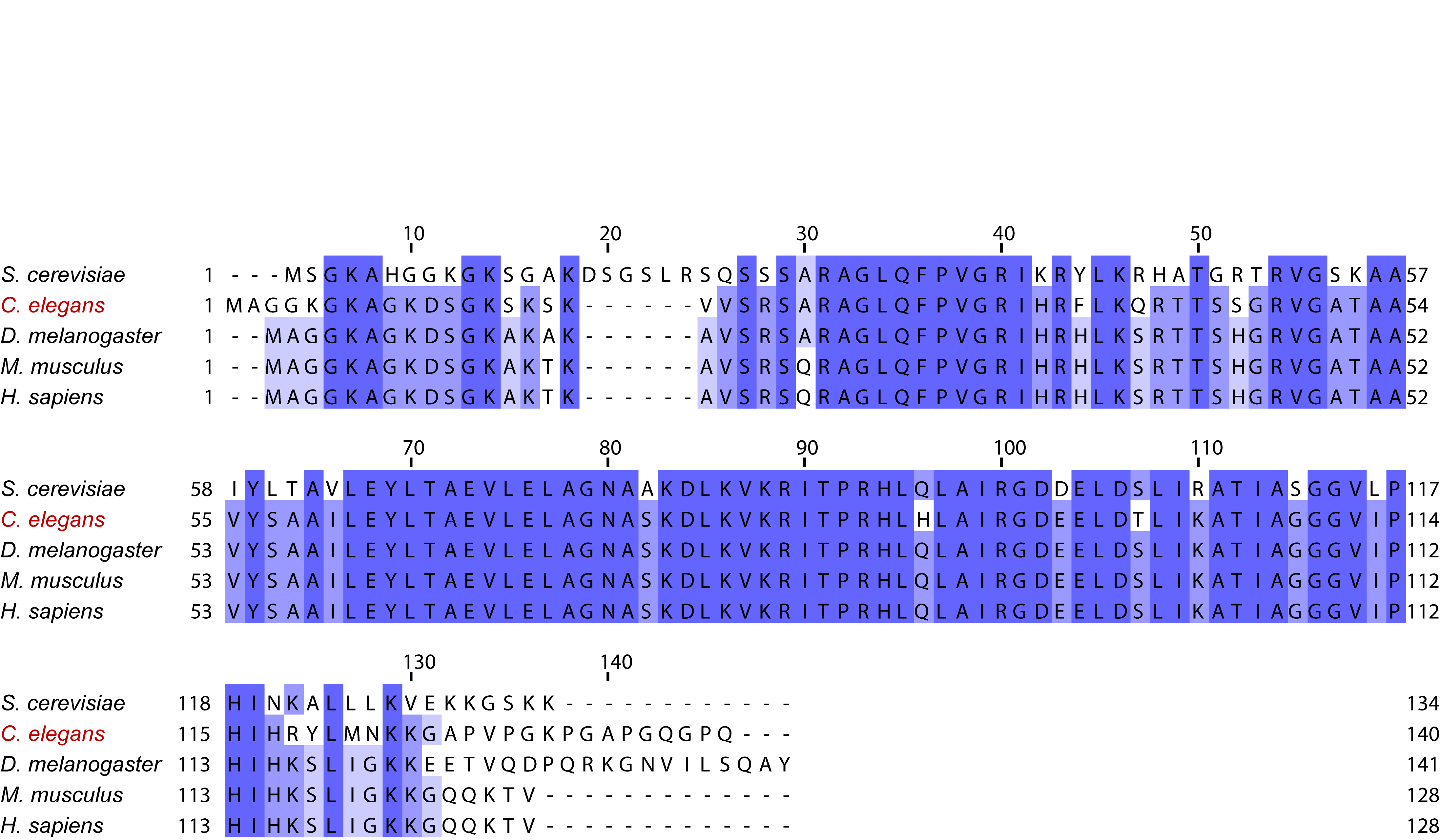

Supplement: Figure S6 — C. elegans HTZ-1 is more similar to Drosophila H2Avd and vertebrate H2A.Z than yeast Htz1. (1.23 MB TIF) [file pgen.1000187.s006.tif]

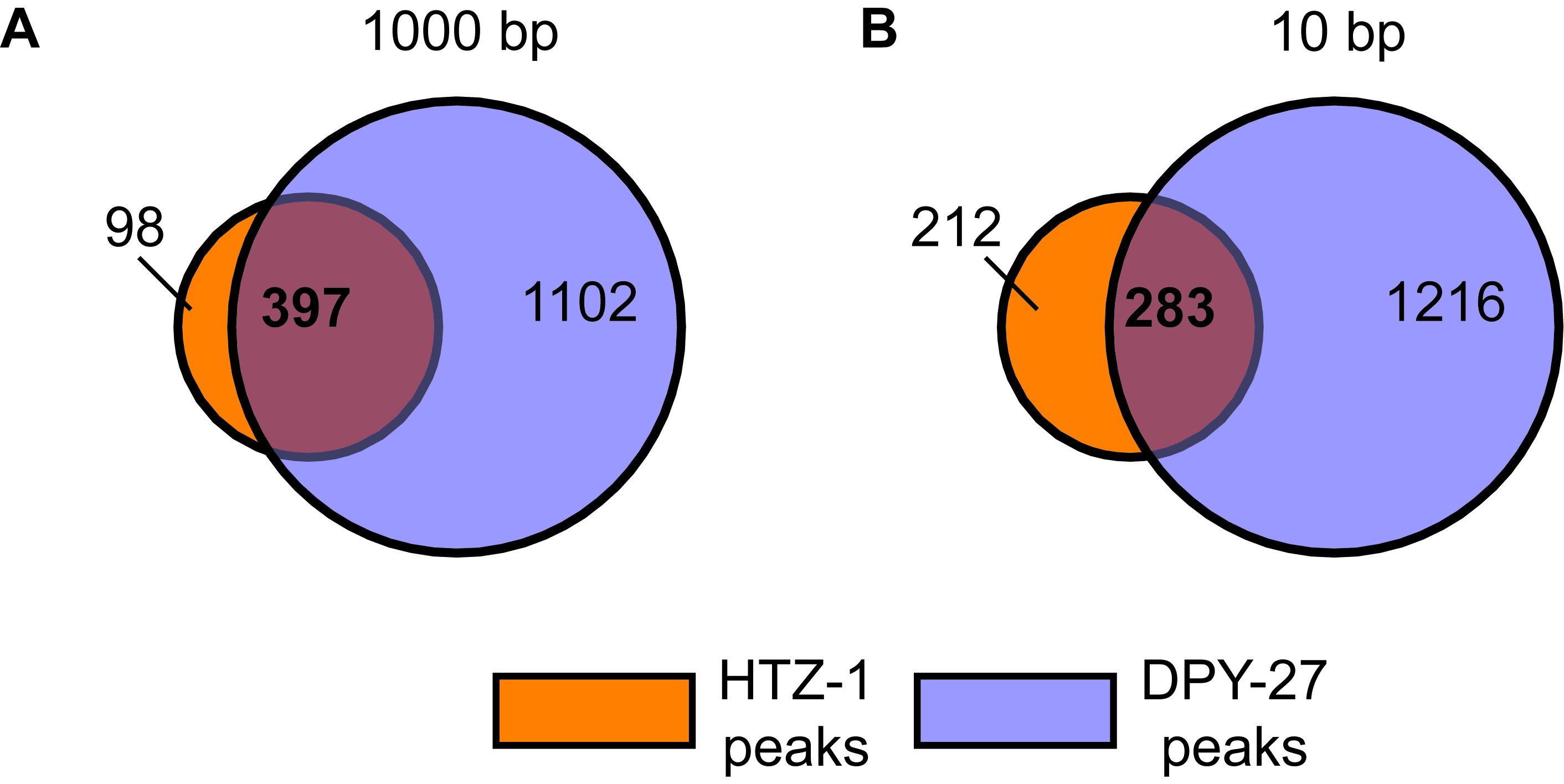

Supplement: Figure S7 — HTZ-1 peaks are coincident with DPY-27 peaks on the X chromosome. (1.26 MB TIF) [file pgen.1000187.s007.tif]

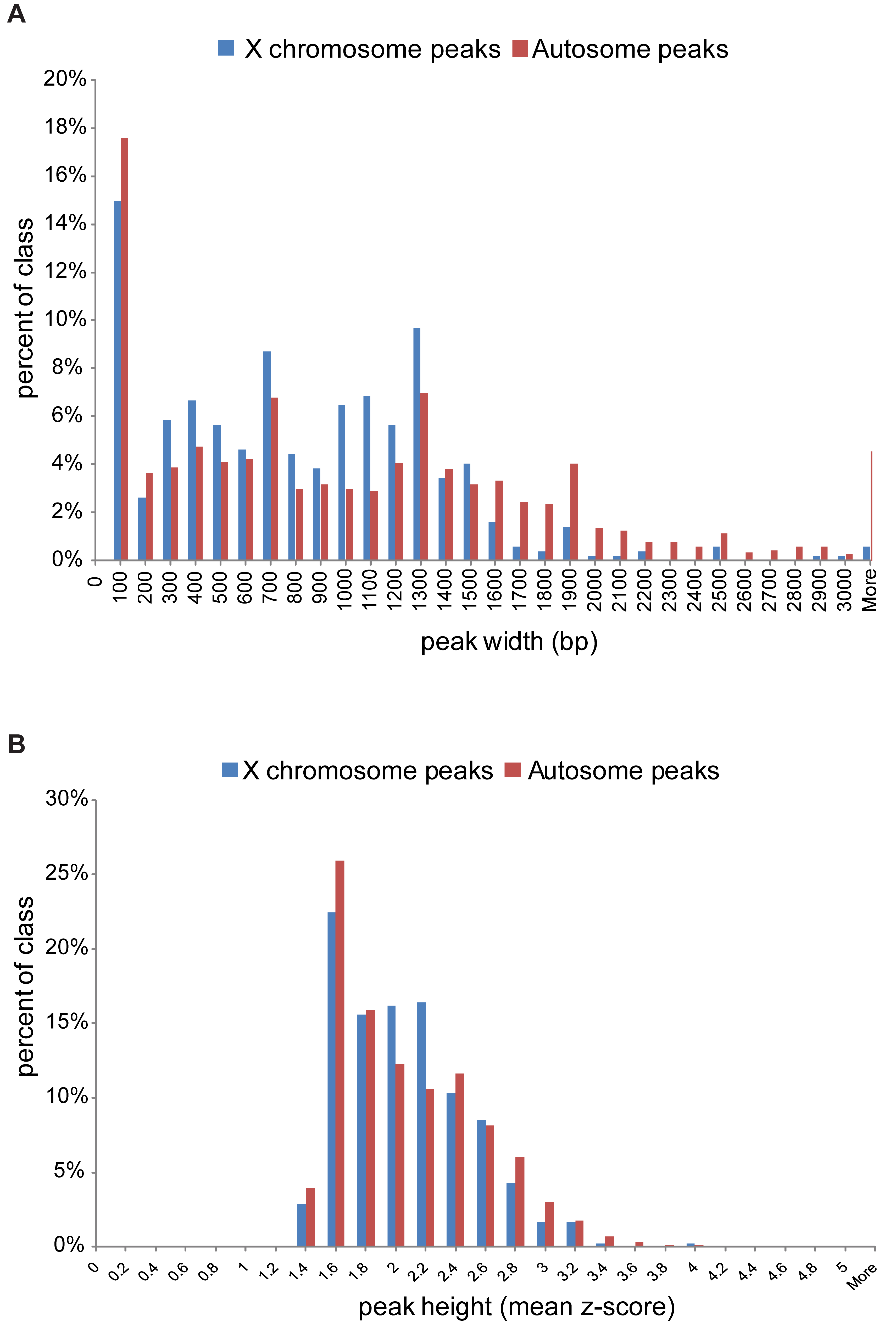

Supplement: Figure S8 — A comparison of mean HTZ-1 peak height and width between X and the autosomes. (1.48 MB TIF) [file pgen.1000187.s008.tif]
