# Supplementary material for: The Genomic Distribution and Function of Histone Variant HTZ-1 during C. elegans Embryogenesis
Source: PLoS Genet. 2008 Sep 12;4(9):e1000187. doi: 10.1371/journal.pgen.1000187 (PMC2522285; doi:10.1371/journal.pgen.1000187)
Supplement: Table S1 — Complete list of all over-represented Gene Ontology Terms. (0.09 MB PDF) [file pgen.1000187.s009.pdf]

GO term	No. of HTZ-1 bound loci annotated to GO term (of 2986)	No. of all loci annotated to GO term (of 22246)	Corrected p-value	Term definition
embryonic development ending in birth or egg hatching	750 (25.12%)	2650 (11.91%)	3.87E-102	The process whose specific outcome is the progression of an embryo over time, from zygote formation until the end of the embryonic life stage. The end of the embryonic life stage is organism specific and may be somewhat arbitrary; for mammals it is usually considered to be birth; for insects the hatching of the first instar larva from the eggshell. As in, but not restricted to, the multicellular animals (Metazoa, ncbi_taxonomy_id:33208).
embryonic development	761 (25.49%)	2744 (12.33%)	6.04E-99	The process whose specific outcome is the progression of an embryo from zygote formation until the end of its embryonic life stage. The end of the embryonic stage is organism-specific and may be somewhat arbitrary. For example, it would be at birth for mammals, larval hatching for insects and seed dormancy in plants.
multicellular organismal development	905 (30.31%)	3541 (15.92%)	6.43E-99	The biological process whose specific outcome is the progression of an organism over time from an initial condition (e.g. a zygote, a young adult or a young single celled organism) to a later condition (e.g. a multicellular animal, an aged adult or a mature single celled organism).
developmental process	938 (31.41%)	3741 (16.82%)	4.22E-98	A biological process whose specific outcome is the progression of an integrated living unit: a cell, tissue, organ, or organism over time from an initial condition to a later condition.
multicellular organismal process	948 (31.75%)	3977 (17.88%)	1.47E-85	The biological processes, occurring at the level of the organism, pertinent to the function of the organism.
reproduction	527 (17.65%)	1893 (8.51%)	3.96E-65	The production by an organism of new individuals that contain some portion of their genetic material inherited from that organism.
growth	553 (18.52%)	2081 (9.35%)	5.09E-61	The increase in size or mass of an entire organism, a part of an organism or a cell.
positive regulation of growth rate	387 (12.96%)	1300 (5.84%)	3.73E-54	Any process that increases the rate of growth of all or part of an organism.
regulation of growth rate	387 (12.96%)	1303 (5.86%)	7.24E-54	Any process that modulates the rate of growth of all or part of an organism.
positive regulation of growth	424 (14.2%)	1530 (6.88%)	2.17E-50	Any process that activates or increases the rate or extent of growth, the increase in size or mass of all or part of an organism.
regulation of growth	433 (14.5%)	1585 (7.12%)	1.00E-49	Any process that modulates the frequency, rate or extent of the growth of all or part of an organism so that it occurs at its proper speed, either globally or in a specific part of the organism's development.

post-embryonic development	459 (15.37%)	1737 (7.81%)	1.34E-48	The process whose specific outcome is the progression of the organism over time, from the completion of embryonic development to the mature structure. See embryonic development.
larval development (sensu Nematoda)	435 (14.57%)	1615 (7.26%)	3.68E-48	The process whose specific outcome is the progression of the larva over time, from its formation to the mature structure. This begins with the newly hatched first-stage larva and ends with the end of the last larval stage (e.g. fourth-stage in C. elegans) before molting to adulthood. As in, but not restricted to, the roundworms (Nematoda, ncbi_taxonomy_id:6231).
larval development	435 (14.57%)	1617 (7.27%)	5.29E-48	The process whose specific outcome is the progression of the larva over time, from its formation to the mature structure. The larva is the early, immature form of any animal when more or less of a metamorphosis takes place, before the assumption of the mature shape.
positive regulation of biological process	445 (14.9%)	1678 (7.54%)	2.17E-47	Any process that activates or increases the frequency, rate or extent of a biological process. Biological processes are regulated by many means; examples include the control of gene expression, protein modification or interaction with a protein or substrate molecule.
sexual reproduction	230 (7.7%)	728 (3.27%)	3.93E-35	The regular alternation, in the life cycle of haplontic, diplontic and diplohaplontic organisms, of meiosis and fertilization which provides for the production offspring. In diplontic organisms there is a life cycle in which the products of meiosis behave directly as gametes, fusing to form a zygote from which the diploid, or sexually reproductive polyploid, adult organism will develop. In diplohaplontic organisms a haploid phase (gametophyte) exists in the life cycle between meiosis and fertilization (e.g. higher plants, many algae and fungi); the products of meiosis are spores that develop as haploid individuals from which haploid gametes develop to form a diploid zygote; diplohaplontic organisms show an alternation of haploid and diploid generations. In haplontic organisms meiosis occurs in the zygote, giving rise to four haploid cells (e.g. many algae and protozoa), only the zygote is diploid and this may form a resistant spore, tiding organisms over hard times.
biological regulation	633 (21.2%)	2964 (13.32%)	5.54E-35	Any process that modulates the frequency, rate or extent of any biological process, quality or function.

metabolic process	942 (31.55%)	4954 (22.27%)	7.60E-34	Processes that cause many of the chemical changes in living organisms, including anabolism and catabolism. Metabolic processes typically transform small molecules, but also include macromolecular processes such as DNA repair and replication, and protein synthesis and degradation.
gamete generation	216 (7.23%)	681 (3.06%)	4.10E-33	The generation and maintenance of gametes.
regulation of biological process	594 (19.89%)	2793 (12.56%)	7.77E-32	Any process that modulates the frequency, rate or extent of a biological process. Biological processes are regulated by many means; examples include the control of gene expression, protein modification or interaction with a protein or substrate molecule.
cellular process	1107 (37.07%)	6138 (27.59%)	3.69E-31	Processes that are carried out at the cellular level, but are not necessarily restricted to a single cell. For example, cell communication occurs among more than one cell, but occurs at the cellular level.
cellular metabolic process	823 (27.56%)	4267 (19.18%)	1.84E-30	The chemical reactions and pathways by which individual cells transform chemical substances.
primary metabolic process	800 (26.79%)	4258 (19.14%)	2.13E-25	Reactions involving those compounds which are formed as a part of the normal anabolic and catabolic processes. These processes take place in most, if not all, cells of the organism.
reproductive process	233 (7.8%)	861 (3.87%)	2.04E-24	A biological process that directly contributes to the process of producing new individuals by one or two organisms. The new individuals inherit some proportion of their genetic material from the parent or parents.
anatomical structure development	296 (9.91%)	1200 (5.39%)	2.91E-24	The biological process whose specific outcome is the progression of an anatomical structure from an initial condition to its mature state. This process begins with the formation of the structure and ends with the mature structure, whatever form that may be including its natural destruction. An anatomical structure is any biological entity that occupies space and is distinguished from its surroundings. Anatomical structures can be macroscopic such as a carpel, or microscopic such as an acrosome.
hermaphrodite genitalia development	158 (5.29%)	502 (2.26%)	3.26E-23	The process whose specific outcome is the progression of the hermaphrodite genitalia over time, from formation to the mature structures.
genitalia development	159 (5.32%)	510 (2.29%)	7.09E-23	The process whose specific outcome is the progression of the genitalia over time, from its formation to the mature structure.
sex differentiation	169 (5.66%)	564 (2.54%)	2.77E-22	The establishment of the sex of an organism by physical differentiation.

organ development	195 (6.53%)	701 (3.15%)	1.29E-21	Development of a tissue or tissues that work together to perform a specific function or functions. Development pertains to the process whose specific outcome is the progression of a structure over time, from its formation to the mature structure. Organs are commonly observed as visibly distinct structures, but may also exist as loosely associated clusters of cells that work together to perform a specific function or functions.
macromolecule metabolic process	670 (22.44%)	3524 (15.84%)	1.41E-21	The chemical reactions and pathways involving macromolecules, large molecules including proteins, nucleic acids and carbohydrates.
reproductive developmental process	173 (5.79%)	593 (2.67%)	2.04E-21	A developmental process by which a progressive change in the state of some part of an organism specifically contributes to its ability to form offspring.
system development	200 (6.7%)	749 (3.37%)	7.14E-20	The process whose specific outcome is the progression of an organismal system over time, from its formation to the mature structure. A system is a regularly interacting or interdependent group of organs or tissues that work together to carry out a given biological process.
biosynthetic process	229 (7.67%)	937 (4.21%)	1.19E-17	The energy-requiring part of metabolism in which simpler substances are transformed into more complex ones, as in growth and other biosynthetic processes.
response to stimulus	388 (12.99%)	1865 (8.38%)	2.00E-17	A change in state or activity of a cell or an organism (in terms of movement, secretion, enzyme production, gene expression, etc.) as a result of a stimulus.
cellular biosynthetic process	195 (6.53%)	771 (3.47%)	1.93E-16	The chemical reactions and pathways resulting in the formation of substances, carried out by individual cells.
cellular component organization and biogenesis	248 (8.31%)	1069 (4.81%)	3.91E-16	The processes involved in the assembly and arrangement of cell structures, including the plasma membrane and any external encapsulating structures such as the cell wall and cell envelope.
establishment of cellular localization	114 (3.82%)	373 (1.68%)	3.72E-15	The directed movement of a substance or cellular entity, such as a protein complex or organelle, to a specific location within, or in the membrane of, a cell.
cellular localization	114 (3.82%)	379 (1.7%)	1.41E-14	The processes by which a substance or cellular entity, such as a protein complex or organelle, is transported to, and/or maintained in, a specific location within or in the membrane of a cell.
locomotory behavior	230 (7.7%)	999 (4.49%)	2.17E-14	The specific movement from place to place of an organism in response to external or internal stimuli. Locomotion of a whole organism in a manner dependent upon some combination of that organism's internal state and external conditions.

nucleobase, nucleoside, nucleotide and nucleic acid metabolic process	338 (11.32%)	1676 (7.53%)	9.70E-13	The chemical reactions and pathways involving nucleobases, nucleosides, nucleotides and nucleic acids.
behavior	263 (8.81%)	1227 (5.52%)	1.35E-12	The specific actions or reactions of an organism in response to external or internal stimuli. Patterned activity of a whole organism in a manner dependent upon some combination of that organism's internal state and external conditions.
protein metabolic process	376 (12.59%)	1936 (8.7%)	7.34E-12	The chemical reactions and pathways involving a specific protein, rather than of proteins in general. Includes protein modification.
biopolymer metabolic process	454 (15.2%)	2436 (10.95%)	1.02E-11	The chemical reactions and pathways involving biopolymers, long, repeating chains of monomers found in nature e.g. polysaccharides and proteins.
translation	88 (2.95%)	290 (1.3%)	3.99E-11	The chemical reactions and pathways resulting in the formation of a protein, rather than of proteins in general.
macromolecule biosynthetic process	111 (3.72%)	404 (1.82%)	4.04E-11	The chemical reactions and pathways resulting in the formation of macromolecules, large molecules including proteins, nucleic acids and carbohydrates.
anatomical structure morphogenesis	175 (5.86%)	760 (3.42%)	1.74E-10	The process by which anatomical structures are generated and organized. Morphogenesis pertains to the creation of form.
cell division	86 (2.88%)	289 (1.3%)	2.62E-10	The processes resulting in the physical partitioning and separation of a cell into daughter cells.
embryonic cleavage	65 (2.18%)	191 (0.86%)	2.67E-10	The first few specialized divisions of an activated animal egg.
cofactor metabolic process	81 (2.71%)	267 (1.2%)	4.16E-10	The chemical reactions and pathways involving a cofactor, a substance that is required for the activity of an enzyme or other protein. Cofactors may be inorganic, such as the metal atoms zinc, iron, and copper in certain forms, or organic, in which case they are referred to as coenzymes. Cofactors may either be bound tightly to active sites or bind loosely with the substrate.
cellular protein metabolic process	355 (11.89%)	1860 (8.36%)	5.58E-10	The chemical reactions and pathways involving a specific protein, rather than of proteins in general, occurring at the level of an individual cell. Includes protein modification.
DNA metabolic process	106 (3.55%)	400 (1.8%)	1.82E-09	The chemical reactions and pathways involving DNA, deoxyribonucleic acid, one of the two main types of nucleic acid, consisting of a long, unbranched macromolecule formed from one, or more commonly, two, strands of linked deoxyribonucleotides.

response to endogenous stimulus	60 (2.01%)	177 (0.8%)	2.63E-09	A change in state or activity of a cell or an organism (in terms of movement, secretion, enzyme production, gene expression, etc.) as a result of an endogenous stimulus.
response to DNA damage stimulus	59 (1.98%)	175 (0.79%)	5.18E-09	A change in state or activity of a cell or an organism (in terms of movement, secretion, enzyme production, gene expression, etc.) as a result of a stimulus indicating damage to its DNA from environmental insults or errors during metabolism.
DNA repair	56 (1.88%)	164 (0.74%)	1.00E-08	The process of restoring DNA after damage. Genomes are subject to damage by chemical and physical agents in the environment (e.g. UV and ionizing radiations, chemical mutagens, fungal and bacterial toxins, etc.) and by free radicals or alkylating agents endogenously generated in metabolism. DNA is also damaged because of errors during its replication. A variety of different DNA repair pathways have been reported that include direct reversal, base excision repair, nucleotide excision repair, photoreactivation, bypass, double-strand break repair pathway, and mismatch repair pathway.
cellular macromolecule metabolic process	360 (12.06%)	1934 (8.69%)	1.15E-08	The chemical reactions and pathways involving macromolecules, large molecules including proteins, nucleic acids and carbohydrates, as carried out by individual cells.
protein transport	74 (2.48%)	258 (1.16%)	8.14E-08	The directed movement of proteins into, out of, within or between cells.
macromolecule localization	86 (2.88%)	319 (1.43%)	8.66E-08	The processes by which a macromolecule is transported to, or maintained in, a specific location.
homeostatic process	83 (2.78%)	304 (1.37%)	9.26E-08	Any of the processes involved in the maintenance of an internal equilibrium within an organism or cell.
osmoregulation	61 (2.04%)	196 (0.88%)	1.00E-07	The control of the osmotic activity of the fluid in a cell, tissue, organ, or organism.
protein localization	85 (2.85%)	315 (1.42%)	1.06E-07	The processes by which a protein is transported to, or maintained in, a specific location.
intracellular transport	68 (2.28%)	230 (1.03%)	1.10E-07	The directed movement of substances within a cell.
establishment of protein localization	77 (2.58%)	276 (1.24%)	1.45E-07	The directed movement of a protein to a specific location.
morphogenesis of an epithelium	72 (2.41%)	252 (1.13%)	1.77E-07	The process by which the anatomical structures of epithelia are generated and organized. Morphogenesis pertains to the creation of form. An epithelium is a sheet of closely packed cells arranged in one or more layers, that covers the outer surfaces of the body or lines any internal cavity or tube.

cell cycle process	66 (2.21%)	225 (1.01%)	2.99E-07	A cellular process that is involved in the progression of biochemical and morphological phases and events that occur in a cell during successive cell replication or nuclear replication events.
regulation of biological quality	90 (3.01%)	353 (1.59%)	7.42E-07	Any process that modulates the frequency, rate or extent of a biological quality. A biological quality is a measurable attribute of an organism or part of an organism, such as size, mass, shape, color, etc.
cell cycle	71 (2.38%)	256 (1.15%)	9.90E-07	The progression of biochemical and morphological phases and events that occur in a cell during successive cell replication or nuclear replication events. Canonically, the cell cycle comprises the replication and segregation of genetic material followed by the division of the cell, but in endocycles or syncytial cells nuclear replication or nuclear division may not be followed by cell division.
chemical homeostasis	65 (2.18%)	228 (1.02%)	1.48E-06	The biological processes involved in the maintenance of an internal equilibrium of a chemical.
vesicle-mediated transport	43 (1.44%)	126 (0.57%)	2.50E-06	The directed movement of substances, either within a vesicle or in the vesicle membrane, into, out of or within a cell.
chromosome segregation	36 (1.21%)	99 (0.45%)	7.19E-06	The process by which genetic material, in the form of chromosomes, is organized and then physically separated and apportioned to two or more sets.
organelle organization and biogenesis	113 (3.78%)	500 (2.25%)	1.09E-05	The assembly and arrangement of any organelle within a cell.
tRNA metabolic process	32 (1.07%)	84 (0.38%)	1.32E-05	The chemical reactions and pathways involving tRNA, transfer RNA, a class of relatively small RNA molecules responsible for mediating the insertion of amino acids into the sequence of nascent polypeptide chains during protein synthesis. Transfer RNA is characterized by the presence of many unusual minor bases, the function of which has not been completely established.
organic acid metabolic process	105 (3.52%)	457 (2.05%)	1.45E-05	The chemical reactions and pathways involving organic acids, any acidic compound containing carbon in covalent linkage.
carboxylic acid metabolic process	105 (3.52%)	457 (2.05%)	1.45E-05	The chemical reactions and pathways involving carboxylic acids, any organic acid containing one or more carboxyl (COOH) groups or anions (COO-).
intracellular protein transport	50 (1.67%)	166 (0.75%)	1.59E-05	The directed movement of proteins in a cell, including the movement of proteins between specific compartments or structures within a cell, such as organelles of a eukaryotic cell.
establishment of organelle localization	36 (1.21%)	102 (0.46%)	1.79E-05	The directed movement of an organelle to a specific location.

response to stress	108 (3.62%)	476 (2.14%)	1.84E-05	A change in state or activity of a cell or an organism (in terms of movement, secretion, enzyme production, gene expression, etc.) as a result of a stimulus indicating the organism is under stress. The stress is usually, but not necessarily, exogenous (e.g. temperature, humidity, ionizing radiation).
DNA replication	27 (0.9%)	65 (0.29%)	2.06E-05	The process whereby new strands of DNA are synthesized. The template for replication can either be DNA or RNA.
organelle localization	36 (1.21%)	104 (0.47%)	3.19E-05	The processes by which an organelle is transported to, and/or maintained in, a specific location.
coenzyme metabolic process	57 (1.91%)	204 (0.92%)	3.23E-05	The chemical reactions and pathways involving coenzymes, any of various nonprotein organic cofactors that are required, in addition to an enzyme and a substrate, for an enzymatic reaction to proceed.
ubiquitin-dependent protein catabolic process	24 (0.8%)	55 (0.25%)	3.98E-05	The chemical reactions and pathways resulting in the breakdown of a protein or peptide by hydrolysis of its peptide bonds, initiated by the covalent attachment of a ubiquitin moiety, or multiple ubiquitin moieties, to the protein.
modification-dependent protein catabolic process	24 (0.8%)	55 (0.25%)	3.98E-05	The chemical reactions and pathways resulting in the breakdown of a protein or peptide by hydrolysis of its peptide bonds, initiated by the covalent modification of the target protein.
modification-dependent macromolecule catabolic process	24 (0.8%)	55 (0.25%)	3.98E-05	The chemical reactions and pathways resulting in the breakdown of a macromolecule, initiated by covalent modification of the target molecule.
proteolysis involved in cellular protein catabolic process	24 (0.8%)	55 (0.25%)	3.98E-05	The hydrolysis of a peptide bond or bonds within a protein during the chemical reactions and pathways resulting in the breakdown of a protein.
protein catabolic process	33 (1.11%)	92 (0.41%)	4.42E-05	The chemical reactions and pathways resulting in the breakdown of a protein by the destruction of the native, active configuration, with or without the hydrolysis of peptide bonds.
cellular protein catabolic process	24 (0.8%)	56 (0.25%)	6.08E-05	The chemical reactions and pathways resulting in the breakdown of a protein by individual cells.
ribonucleotide metabolic process	29 (0.97%)	79 (0.36%)	0.0001608	The chemical reactions and pathways involving a ribonucleotide, a compound consisting of ribonucleoside (a base linked to a ribose sugar) esterified with a phosphate moiety at either the 3' or 5'-hydroxyl group of its glycose moiety.

purine nucleotide metabolic process	28 (0.94%)	77 (0.35%)	0.0003288	The chemical reactions and pathways involving a purine nucleotide, a compound consisting of nucleoside (a purine base linked to a deoxyribose or ribose sugar) esterified with a phosphate moiety at either the 3' or 5'-hydroxyl group of its glycose moiety.
multicellular organism growth	104 (3.48%)	479 (2.15%)	0.0003373	The increase in size or mass of an entire organism, as opposed to cell growth.
regulation of body size	104 (3.48%)	479 (2.15%)	0.0003373	Any process that modulates the frequency, rate or extent of growth of the body of an organism so that it reaches its usual body size.
cytokinesis	38 (1.27%)	123 (0.55%)	0.0003959	The processes resulting in the division of the cytoplasm of a cell and its separation into two daughter cells. Cytokinesis usually occurs after growth, replication, and segregation of cellular components.
regulation of vulval development (sensu Nematoda)	30 (1%)	87 (0.39%)	0.0004808	Any process that modulates the frequency, rate or extent of development of the vulva. As in, but not restricted to, the roundworms (Nematoda, ncbi_taxonomy_id:6231).
purine ribonucleotide metabolic process	27 (0.9%)	75 (0.34%)	0.0006698	The chemical reactions and pathways involving a purine ribonucleotide, a compound consisting of ribonucleoside (a purine base linked to a ribose sugar) esterified with a phosphate moiety at either the 3' or 5'-hydroxyl group of its glycose moiety.
RNA processing	42 (1.41%)	146 (0.66%)	0.0008584	Any process involved in the conversion of a primary RNA transcript into one or more mature RNA molecules.
secretory pathway	21 (0.7%)	51 (0.23%)	0.0009068	The pathway along which proteins and other substances are moved around and out of the cell. After synthesis on the ribosomes of the endoplasmic reticulum (ER), completed polypeptide chains are moved to the Golgi complex and subsequently sorted to various destinations. Proteins synthesized and sorted in the secretory pathway include not only those that are secreted from the cell but also enzymes and other resident proteins in the lumen of the ER, Golgi, and lysosomes as well as integral proteins in the membranes of these organelles and the plasma membrane.
cellular developmental process	81 (2.71%)	356 (1.6%)	0.0009543	A biological process whose specific outcome is the progression of a cell over time from an initial condition to a later condition.

cell differentiation	80 (2.68%)	353 (1.59%)	0.0013105	The process whereby relatively unspecialized cells, e.g. embryonic or regenerative cells, acquire specialized structural and/or functional features that characterize the cells, tissues, or organs of the mature organism or some other relatively stable phase of the organism's life history. Differentiation includes the processes involved in commitment of a cell to a specific fate.
positive regulation of body size	89 (2.98%)	407 (1.83%)	0.0017009	Any process that activates or increases the frequency, rate or extent of growth of an organism to reach its usual body size.
tRNA aminoacylation for protein translation	20 (0.67%)	49 (0.22%)	0.0019411	The synthesis of aminoacyl tRNA by the formation of an ester bond between the 3'-hydroxyl group of the most 3' adenosine of the tRNA, to be used in ribosome-mediated polypeptide synthesis.
amino acid activation	20 (0.67%)	49 (0.22%)	0.0019411	The modification of an amino acid to an active form, for incorporation into a peptide, protein or other macromolecule.
tRNA aminoacylation	20 (0.67%)	49 (0.22%)	0.0019411	The synthesis of aminoacyl tRNA by the formation of an ester bond between the 3'-hydroxyl group of the most 3' adenosine of the tRNA, usually catalyzed by the cognate aminoacyl-tRNA ligase. A given aminoacyl-tRNA ligase aminoacylates all species of an isoaccepting group of tRNA molecules.
DNA-dependent DNA replication	14 (0.47%)	27 (0.12%)	0.0021803	The process whereby new strands of DNA are synthesized, using parental DNA as a template for the DNA-dependent DNA polymerases that synthesize the new strands.
cell cycle phase	32 (1.07%)	102 (0.46%)	0.0021987	A cell cycle process comprising the steps by which a cell progresses through one of the biochemical and morphological phases and events that occur during successive cell replication or nuclear replication events.
meiotic cell cycle	26 (0.87%)	75 (0.34%)	0.0024444	Progression through the phases of the meiotic cell cycle, in which canonically a cell replicates to produce four offspring with half the chromosomal content of the progenitor cell.
macromolecule catabolic process	54 (1.81%)	214 (0.96%)	0.0024688	The chemical reactions and pathways resulting in the breakdown of a macromolecule, any large molecule including proteins, nucleic acids and carbohydrates.
nucleoside monophosphate metabolic process	13 (0.44%)	24 (0.11%)	0.0026651	The chemical reactions and pathways involving a nucleoside monophosphate, a glycosamine consisting of a base linked to a deoxyribose or ribose sugar esterified with phosphate on its glycose moiety.

nucleoside monophosphate biosynthetic process	13 (0.44%)	24 (0.11%)	0.0026651	The chemical reactions and pathways resulting in the formation of a nucleoside monophosphate, a glycosamine consisting of a base linked to a deoxyribose or ribose sugar esterified with phosphate on its glycose moiety.
meiosis	25 (0.84%)	71 (0.32%)	0.0027462	Progression through meiosis, the specialized nuclear and cell division in which a single diploid cell undergoes two nuclear divisions following a single round of DNA replication in order to produce four daughter cells that contain half the number of chromosomes as the diploid cell. Meiosis occurs during the formation of gametes from diploid organisms and at the beginning of haplophase in those organisms that alternate between diploid and haploid generations.
M phase of meiotic cell cycle	25 (0.84%)	72 (0.32%)	0.0036738	Progression through M phase, the part of the meiotic cell cycle during which meiosis takes place.
cell development	65 (2.18%)	277 (1.25%)	0.003702	The process whose specific outcome is the progression of the cell over time, from its formation to the mature structure. Cell development does not include the steps involved in committing a cell to a specific fate.
vulval development (sensu Nematoda)	37 (1.24%)	130 (0.58%)	0.004951	The process whose specific outcome is the progression of the vulva over time, from its formation to the mature structure. Vulval development begins during the larval stages to give rise to a fully formed vulva in the adult. As in, but not restricted to, the roundworms (Nematoda, ncbi_taxonomy_id:6231).
ribonucleoside monophosphate biosynthetic process	12 (0.4%)	22 (0.1%)	0.0059387	The chemical reactions and pathways resulting in the formation of a ribonucleoside monophosphate, a glycosamine consisting of a base linked to a ribose sugar esterified with phosphate on its glycose moiety
ribonucleoside monophosphate metabolic process	12 (0.4%)	22 (0.1%)	0.0059387	The chemical reactions and pathways involving a ribonucleoside monophosphate, a glycosamine consisting of a base linked to a ribose sugar esterified with phosphate on its glycose moiety.
amino acid metabolic process	56 (1.88%)	231 (1.04%)	0.0060651	The chemical reactions and pathways involving amino acids, organic acids containing one or more amino substituents.
cofactor biosynthetic process	45 (1.51%)	173 (0.78%)	0.0070786	The chemical reactions and pathways resulting in the formation of a cofactor, a substance that is required for the activity of an enzyme or other protein.

nucleotide metabolic process	44 (1.47%)	168 (0.76%)	0.0073654	The chemical reactions and pathways involving a nucleotide, a nucleoside that is esterified with (ortho)phosphate or an oligophosphate at any hydroxyl group on the glycose moiety; may be mono-, di- or triphosphate; this definition includes cyclic-nucleotides (nucleoside cyclic phosphates).
cytoskeleton organization and biogenesis	54 (1.81%)	222 (1%)	0.0079366	A process that is carried out at the cellular level which results in the formation, arrangement of constituent parts, or disassembly of cytoskeletal structures.
DNA recombination	20 (0.67%)	53 (0.24%)	0.0081162	The processes by which a new genotype is formed by reassortment of genes resulting in gene combinations different from those that were present in the parents. In eukaryotes genetic recombination can occur by chromosome assortment, intrachromosomal recombination, or nonreciprocal interchromosomal recombination. Intrachromosomal recombination occurs by crossing over. In bacteria it may occur by genetic transformation, conjugation, transduction, or F-duction.
gastrulation with mouth forming first	19 (0.64%)	49 (0.22%)	0.0085779	Gastrulation, as in, but not restricted to, the taxon Protostomia (Protostomia, ncbi_taxonomy_id:33317).
gastrulation	24 (0.8%)	71 (0.32%)	0.0096908	A complex and coordinated series of cellular movements that occurs at the end of cleavage during embryonic development of most animals. The details of gastrulation vary from species to species, but usually result in the formation of the three primary germ layers, ectoderm, mesoderm and endoderm.
