# Supplementary material for: The Genomic Distribution and Function of Histone Variant HTZ-1 during C. elegans Embryogenesis
Source: PLoS Genet. 2008 Sep 12;4(9):e1000187. doi: 10.1371/journal.pgen.1000187 (PMC2522285; doi:10.1371/journal.pgen.1000187)
Supplement: Table S2 — The relationship between operons, operon genes, and HTZ-1 occupancy at promoters. (0.10 MB PDF) [file pgen.1000187.s010.pdf]

Operon	Operon Gene enriched with HTZ-1	Huang *et al*[1] Operon	Huang *et al*[1] Internal Promoter Gene
CEOP1008	W04C9.4		
CEOP1012	F56A6.4		
CEOP1016	Y48G8AL.7		
CEOP1017	Y48G8AL.5, Y48G8AL.15		
CEOP1020	Y71G12B.14, Y71G12B.15		
CEOP1024	C53H9.1, C53H9.2		
CEOP1032	Y37E3.8		
CEOP1036	Y37E3.9		
CEOP1040	Y39G10AR.20, Y39G10AR.9		
CEOP1044	F23C8.6		
CEOP1048	Y74C10AR.1		
CEOP1052	Y71F9B.6		
CEOP1056	Y71F9B.3		
CEOP1064	Y71F9AL.13		
CEOP1068	Y71F9AL.8, Y71F9AL.9		
CEOP1072	Y71F9AL.16		
CEOP1076	Y71F9AM.5, Y71F9AL.1		
CEOP1080	Y54E10BR.4		
CEOP1084	M01B12.5		
CEOP1088	Y54E10A.7		
CEOP1096	Y47G6A.11, Y47G6A.28		
CEOP1100	Y47G6A.24, Y47G6A.25		
CEOP1104	T12F5.2, T12F5.3		
CEOP1112	C32E8.10		
CEOP1116	F53F10.4		
CEOP1120	T03F1.8, T03F1.9		
CEOP1124	T03F1.3		
CEOP1152	C18E3.6, C18E3.7		
CEOP1160	C43E11.8		
CEOP1164	C43E11.12		
CEOP1176	ZK973.10		
CEOP1180	C41D11.9, C41D11.7		
CEOP1184	D1007.6		
CEOP1188	D1007.10, D1007.12		
CEOP1192	D1007.16		
CEOP1196	Y119C1A.1		
CEOP1200	T04D1.3		
CEOP1204	F57C9.4, F57C9.5		
CEOP1208	F28B3.7		
CEOP1216	Y110A7A.14		
CEOP1220	Y110A7A.6, Y110A7A.5, Y110A7A.8		
CEOP1224	F56A3.3		
CEOP1228	B0261.1, B0261.4		
CEOP1232	C01G8.3, C01G8.5	CEOP1232	
CEOP1236	F48C1.6		
CEOP1240	F55A12.3		
CEOP1248	F27C1.6, C30H7.2, C24A11.9		
CEOP1252	C09D4.4		
CEOP1264	F46F11.9, F46F11.4, F46F11.6		
CEOP1268	F46F11.7		
CEOP1272	F46F11.8		
CEOP1276	F55F8.4, F55F8.6		
CEOP1280	T19B4.5		
CEOP1284	F33D11.12, F33D11.11		
CEOP1288	C06A5.1		
CEOP1292	C27A12.3		
CEOP1296	C27A12.9, C27A12.8, C27A12.7, C27A12.6, ZK484.4	CEOP1296	C27A12.8
CEOP1300	T27A3.6, T27A3.7		
CEOP1304	T09B4.9		
CEOP1308	T05E7.3, T09B4.2		
CEOP1312	T08B2.9		

[1]Huang P, Pleasance ED, Maydan JS, Hunt-Newbury R, O'Neil NJ, et al. (2007) Identification and analysis of internal promoters in Caenorhabditis elegans operons. Genome Res 17: 1478-1485.

CEOP1316	C48E7.11		
CEOP1324	C55B7.9		
CEOP1326	T10E9.7		
CEOP1332	F57B10.14		
CEOP1336	F57B10.6, F57B10.7		
CEOP1340	F57B10.12		
CEOP1344	W02D3.2		
CEOP1348	W02D3.9, W02D3.10, W02D3.11	CEOP1348	W02D3.9
CEOP1352	C37A2.7		
CEOP1356	C48B6.3		
CEOP1358	T10B11.3		
CEOP1360	T10B11.6, T10B11.7		
CEOP1364	F22D6.2		
CEOP1368	K07G5.6, K07G5.1		
CEOP1372	C01H6.6, C01H6.5		
CEOP1376	C01H6.9		
CEOP1380	R06C7.7		
CEOP1384	F21C3.5		
CEOP1388	F13G3.5, F13G3.6		
CEOP1394	F13G3.10		
CEOP1396	ZK524.3, ZK524.4		
CEOP1400	T25G3.4, T25G3.2, T25G3.1		
CEOP1404	D2030.4		
CEOP1408	D2030.7		
CEOP1412	F29D11.2, F26A3.8, F26A3.2, F26A3.3		
CEOP1416	T23G11.7, T23G11.4, T24B1.1		
CEOP1424	F30F8.3		
CEOP1428	F30F8.8		
CEOP1432	F20G4.1, F20G4.3		
CEOP1436	T22C1.6, T22C1.5, T22C1.1	CEOP1436	T22C1.6
CEOP1448	C54G4.9, K04G2.1		
CEOP1452	F18C12.2		
CEOP1456	T01G9.6, T01G9.4		
CEOP1460	F52B5.3		
CEOP1464	C34B7.4, C34B7.2, C34B7.1		
CEOP1468	F16D3.4		
CEOP1472	T19A6.2		
CEOP1476	F02E9.10		
CEOP1480	K02B12.8		
CEOP1484	R05D11.4, R05D11.3		
CEOP1492	F43G9.12		
CEOP1496	F39H2.3		
CEOP1500	K07A12.7		
CEOP1504	F36A2.1, F36A2.4		
CEOP1508	T08G11.4, T08G11.1		
CEOP1512	F32H2.1		
CEOP1516	F36F2.4, F36F2.3		
CEOP1520	F32H2.10		
CEOP1528	W06D4.5		
CEOP1532	W10D5.2		
CEOP1536	ZK858.6		
CEOP1540	F25H5.5		
CEOP1542	F36H2.2		
CEOP1544	C17E4.10, C17E4.6		
CEOP1548	K07A1.1		
CEOP1552	K07A1.9, K07A1.11		
CEOP1556	T05F1.6		
CEOP1560	C16C2.3		
CEOP1564	F26E4.8		
CEOP1566	K10C3.5		
CEOP1568	Y106G6A.1		
CEOP1572	T24D1.5		
CEOP1576	T23D8.4		
CEOP1580	T23D8.7		

[1]Huang P, Pleasance ED, Maydan JS, Hunt-Newbury R, O'Neil NJ, et al. (2007) Identification and analysis of internal promoters in Caenorhabditis elegans operons. Genome Res 17: 1478-1485.

CEOP1584	F10G8.6, F10G8.3		
CEOP1588	C25A1.7, C25A1.5		
CEOP1592	Y106G6E.6		
CEOP1596	ZC434.5		
CEOP1600	F45H11.3		
CEOP1604	Y106G6H.5		
CEOP1608	Y106G6H.7		
CEOP1612	Y106G6H.12		
CEOP1616	F59C6.4		
CEOP1620	F25H2.6		
CEOP1624	F25H2.13		
CEOP1628	C43H8.2		
CEOP1632	B0511.13, B0511.10		
CEOP1636	C34B2.6, C34B2.7		
CEOP1640	B0205.8		
CEOP1644	B0205.7		
CEOP1648	F55A3.2, F55A3.3		
CEOP1650	F49D11.1		
CEOP1652	F56G4.4		
CEOP1660	R06C1.1, R06C1.2		
CEOP1664	C15C6.3		
CEOP1668	T27F6.5, T27F6.4		
CEOP1672	T26E3.4		
CEOP1680	F08A8.7, C47B2.2, C47B2.1		
CEOP1682	CC4.3		
CEOP1684	F22G12.6		
CEOP1688	M01E5.3		
CEOP1692	W08E3.1		
CEOP1696	C01A2.5		
CEOP1700	Y40B1B.8		
CEOP1704	Y87G2A.8, Y87G2A.7		
CEOP1712	Y87G2A.10, Y6B3A.1, W09C5.1		
CEOP1716	Y6B3B.9, Y6B3B.5		
CEOP1724	W09G3.6, W09G3.3, W09G3.1, W04A8.1		
CEOP1728	W04A8.6		
CEOP1732	Y105E8A.13		
CEOP1736	Y105E8A.20		
CEOP1740	Y105E8A.23, Y105E8A.25		
CEOP1744	Y105E8B.6, Y105E8B.3, Y105E8B.4	CEOP1744	Y105E8B.3
CEOP1748	Y54E5A.6		
CEOP1756	K05C4.7		
CEOP1760	F39B2.7		
CEOP1764	F39B2.2		
CEOP1772	F33H2.6, F33H2.1		
CEOP1776	F31C3.3, F31C3.4	CEOP1776	
CEOP1780	C10H11.9		
CEOP1782	C32E8.2, C32E8.3		
CEOP1786	C47B2.6		
CEOP1890	DY3.1		
CEOP1892	E01A2.5, E01A2.6		
CEOP1896	F28D9.1		
CEOP1900	F55A3.1		
CEOP1908	Y34D9A.3		
CEOP1910	Y39G10AR.12, Y39G10AR.13		
CEOP1916	Y54E10A.10, Y54E10A.11		
CEOP192	C17E4.5		
CEOP1926	F37D6.1		
CEOP1927	F28B3.5		
CEOP1929	D1081.8		
CEOP1930	F26E4.11, F26E4.9		
CEOP1931	Y51F10.5		
CEOP1932	F16A11.3, F16A11.2, F16A11.1		
CEOP1935	ZC308.4		
CEOP1936	Y53C10A.14		

[1]Huang P, Pleasance ED, Maydan JS, Hunt-Newbury R, O'Neil NJ, et al. (2007) Identification and analysis of internal promoters in Caenorhabditis elegans operons. Genome Res 17: 1478-1485.

CEOP1937	T01H8.2, T01H8.1		
CEOP1938	Y23H5A.3		
CEOP1939	Y48G8AL.10, Y48G8AL.14		
CEOP1940	Y48G1C.9, Y48G1C.1		
CEOP2004	C23H3.4		
CEOP2016	Y43H11AL.3		
CEOP2020	B0432.10		
CEOP2024	B0432.2, B0432.4		
CEOP2028	W10D9.5		
CEOP2032	T02H6.2		
CEOP2044	F56D12.1		
CEOP2048	Y51H7C.7		
CEOP2052	F52C6.8, F52C6.11		
CEOP2056	F52C6.1, F52C6.4		
CEOP2060	F59H6.10		
CEOP2068	F54A3.6		
CEOP2070	F53G2.7		
CEOP2072	Y110A2AL.14		
CEOP2074	Y110A2AM.3		
CEOP2076	Y110A2AR.2, Y110A2AR.3		
CEOP2084	Y25C1A.8		
CEOP2108	Y49F6B.2		
CEOP2110	F54D10.5		
CEOP2116	C16A11.3, C16A11.4		
CEOP2120	C01F1.2		
CEOP2124	H20J04.4, H20J04.6		
CEOP2128	W06B4.2		
CEOP2136	C33F10.4, F26G1.1		
CEOP2140	R05F9.9, R05F9.10		
CEOP2148	Y38A8.2, Y38A8.3		
CEOP2152	T27F7.1		
CEOP2156	F33G12.3, F33G12.5		
CEOP2160	C04G6.4		
CEOP2168	F09E5.17		
CEOP2172	F09E5.3		
CEOP2176	EEED8.9, EEED8.8	CEOP2176	
CEOP2182	F07F6.8, F07F6.4		
CEOP2184	F56D1.3		
CEOP2188	ZK177.5, ZK177.6		
CEOP2192	C17G10.2		
CEOP2200	F59E12.2		
CEOP2204	C25H3.12		
CEOP2208	C25H3.7		
CEOP2212	C25H3.14, C25H3.4		
CEOP2216	C18A3.3		
CEOP2224	ZK1248.14		
CEOP2228	ZK1248.16		
CEOP2232	F59G1.7, F28B12.3		
CEOP2236	C29H12.2		
CEOP2238	T24H7.2, T24H7.1, T24H7.4		
CEOP2240	C32D5.9, C32D5.11		
CEOP2244	K10B2.4		
CEOP2256	C56C10.13		
CEOP2260	T14B4.1, F41G3.14, T14B4.3		
CEOP2264	C44B7.2		
CEOP2268	C15F1.4		
CEOP2272	C15F1.5, C15F1.7		
CEOP2276	C52E12.4		
CEOP2284	T02G5.8		
CEOP2288	F10E7.8		
CEOP2292	C30G12.6, C30G12.7		
CEOP2296	F45E12.3		
CEOP2298	F43E2.1		
CEOP2300	F18A1.4		

[1]Huang P, Pleasance ED, Maydan JS, Hunt-Newbury R, O'Neil NJ, et al. (2007) Identification and analysis of internal promoters in Caenorhabditis elegans operons. Genome Res 17: 1478-1485.

CEOP2308	B0495.9		
CEOP2316	T01H3.2		
CEOP2320	ZK675.2, ZK675.1		
CEOP2328	C08B11.7		
CEOP2332	T05H10.2		
CEOP2336	T05H10.5		
CEOP2344	F22B5.2		
CEOP2348	F22B5.9, F22B5.7		
CEOP2352	M05D6.6		
CEOP2356	T13H5.7		
CEOP2364	F54C9.5		
CEOP2368	F54C9.9		
CEOP2372	F28C6.6		
CEOP2376	D2085.2		
CEOP2384	C34C6.4, C34C6.5		
CEOP2388	T01B7.4		
CEOP2390	T01B7.5		
CEOP2396	T21B10.2, T21B10.7		
CEOP2404	C18E9.11		
CEOP2408	T24B8.7		
CEOP2412	T24H10.3		
CEOP2416	T23G7.2, T23G7.4		
CEOP2420	ZK1067.3, ZK1067.2		
CEOP2424	D2013.8		
CEOP2436	E02H1.3		
CEOP2440	ZK1307.5		
CEOP2444	ZK1320.12, ZK1320.11		
CEOP2448	Y53C12A.3		
CEOP2450	Y53C12A.1, Y53C12A.6		
CEOP2452	Y53C12B.1		
CEOP2468	C05C10.6		
CEOP2472	T01E8.5		
CEOP2476	ZK970.2		
CEOP2484	T09F3.5, T09F3.3		
CEOP2488	ZK673.3, ZK673.2		
CEOP2492	R166.4, R166.5	CEOP2492	
CEOP2496	C06A1.5		
CEOP2500	M28.6, M28.5		
CEOP2504	R06F6.5		
CEOP2508	F59E10.3		
CEOP2512	F33A8.5		
CEOP2516	C09H10.6		
CEOP2520	F40F8.3, F40F8.1, F40F8.9	CEOP2520	F40F8.1
CEOP2524	T06D8.6, T06D8.8		
CEOP2528	C47G2.5		
CEOP2532	B0491.1		
CEOP2536	VW02B12L.4, VW02B12L.3		
CEOP2540	W02B12.7		
CEOP2544	B0334.11		
CEOP2548	B0334.4		
CEOP2552	Y62F5A.1		
CEOP2556	F54D5.11		
CEOP2564	D1043.1, F54D5.14		
CEOP2572	VF13D12L.3, C47D12.8	CEOP2572	C47D12.8
CEOP2576	F43G6.9		
CEOP2580	W03C9.5		
CEOP2584	Y17G7B.2		
CEOP2588	Y17G7B.9, Y17G7B.7		
CEOP2590	Y43F11A.5		
CEOP2592	Y57A10A.7, Y57A10A.5, Y57A10A.3, Y57A10A.1		
CEOP2600	Y46G5A.5		
CEOP2601	F15D4.1		
CEOP2606	Y48C3A.20		
CEOP2620	Y51H1A.6, Y51H1A.3		

[1]Huang P, Pleasance ED, Maydan JS, Hunt-Newbury R, O'Neil NJ, et al. (2007) Identification and analysis of internal promoters in Caenorhabditis elegans operons. Genome Res 17: 1478-1485.

CEOP2624	W01G7.5, W01G7.3		
CEOP2640	R06A4.9		
CEOP2644	F57C2.2, F57C2.1		
CEOP2648	Y54E2A.2, Y54E2A.3		
CEOP2652	R05H10.3		
CEOP2656	Y53F4B.13, Y53F4B.12		
CEOP2660	Y53F4B.21		
CEOP2666	F10E7.5		
CEOP2668	F18A1.6		
CEOP2672	F23F1.8		
CEOP2686	W10D9.4		
CEOP2691	F11G11.5, F11G11.7		
CEOP2692	C07E3.1		
CEOP2693	C14A4.11		
CEOP2694	C47G2.4		
CEOP2695	Y57A10A.29		
CEOP2696	F52H3.1		
CEOP2697	Y48B6A.3		
CEOP2698	Y17G7B.12, Y17G7B.6		
CEOP2699	Y17G7B.13		
CEOP2700	Y48B6A.12		
CEOP2702	T09A5.15		
CEOP2703	F41C3.4		
CEOP2705	ZK669.4		
CEOP3012	C29F9.2		
CEOP3016	F40G9.1, F40G9.3		
CEOP3020	Y50D7A.3, Y50D7A.2		
CEOP3024	F10C5.1, F10C5.2		
CEOP3028	T19C3.7, W06E11.5		
CEOP3034	F42G9.6		
CEOP3044	Y6D11A.2, K02F3.1	CEOP3044	Y6D11A.2
CEOP3048	F23H11.4, F23H11.3		
CEOP3052	Y92C3B.2, Y92C3B.3		
CEOP3056	F58B6.3, Y92C3B.1		
CEOP3061	Y82E9BR.14, Y82E9BR.15		
CEOP3062	M01G5.5		
CEOP3068	Y39A3CL.7		
CEOP3072	Y39A3CR.4		
CEOP3076	W04B5.5		
CEOP3080	Y71H2B.5, Y71H2B.3, Y71H2B.6		
CEOP3086	Y71H2AM.2		
CEOP3088	H06I04.4		
CEOP3100	R148.3		
CEOP3108	F59A2.4, F59A2.3		
CEOP3112	F59A2.5		
CEOP3120	C34C12.8		
CEOP3124	M01F1.8, M01F1.3	CEOP3124	M01F1.3
CEOP3128	C46F11.5, C46F11.4		
CEOP3132	T27D1.1, C14B1.1		
CEOP3136	C14B1.6, C14B1.10, C14B1.4		
CEOP3140	C14B1.8		
CEOP3144	ZK1058.4		
CEOP3148	T02C12.2		
CEOP3156	C16C10.5		
CEOP3160	R74.1		
CEOP3164	T08A11.2		
CEOP3168	R10E4.9, H38K22.1		
CEOP3172	H38K22.2		
CEOP3174	B0285.4		
CEOP3176	B0285.8		
CEOP3184	R07E5.13, R07E5.1		
CEOP3188	R07E5.7		
CEOP3192	C07G2.3		
CEOP3196	F35G12.5, F35G12.4		

[1]Huang P, Pleasance ED, Maydan JS, Hunt-Newbury R, O'Neil NJ, et al. (2007) Identification and analysis of internal promoters in Caenorhabditis elegans operons. Genome Res 17: 1478-1485.

CEOP3200	F35G12.8		
CEOP3204	F35G12.9, F35G12.10		
CEOP3212	T04A8.13		
CEOP3216	T04A8.14		
CEOP3220	C38D4.5		
CEOP3224	C35D10.7, F26A1.1	CEOP3224	
CEOP3228	C35D10.16		
CEOP3232	F26F4.6, F26F4.5, F26F4.7	CEOP3232	
CEOP3240	F26F4.11, C26E6.11, C26E6.9		
CEOP3244	C26E6.6	CEOP3244	
CEOP3252	R144.7		
CEOP3256	R144.3, R144.4		
CEOP3260	T10F2.1	CEOP3260	
CEOP3264	K10D2.5		
CEOP3268	C34E10.5, C34E10.6		
CEOP3272	C34E10.3		
CEOP3280	ZC395.3		
CEOP3284	F52C9.7		
CEOP3292	Y32H12A.2		
CEOP3304	F48E8.4, F48E8.5	CEOP3304	
CEOP3310	F56D2.7, F56D2.1		
CEOP3312	C05D2.6		
CEOP3314	F54E7.8		
CEOP3316	F54E7.1		
CEOP3320	B0336.5		
CEOP3324	B0336.8		
CEOP3328	B0336.10		
CEOP3332	B0336.2	CEOP3332	B0336.2
CEOP3336	F01F1.7, F01F1.8		
CEOP3340	F01F1.5, F01F1.6		
CEOP3344	F01F1.11		
CEOP3348	F01F1.2		
CEOP3350	C28H8.3, C28H8.4		
CEOP3352	F25B5.6		
CEOP3360	T12A2.8		
CEOP3364	C16A3.6, C16A3.7		
CEOP3368	C16A3.3		
CEOP3372	C05D11.10		
CEOP3376	C05D11.3		
CEOP3384	C05D11.13, T26A5.7	CEOP3384	
CEOP3388	T26A5.5		
CEOP3390	T26A5.2, T26A5.3		
CEOP3392	F20H11.1, F20H11.6		
CEOP3396	Y40D12A.1		
CEOP3404	F57B9.4, F57B9.6		
CEOP3408	ZK418.6		
CEOP3412	K04G7.11		
CEOP3416	F37C12.3, F37C12.1, F37C12.4		
CEOP3420	R151.9		
CEOP3424	R151.8, T20H4.5		
CEOP3425	F56C9.10		
CEOP3426	B0361.8		
CEOP3428	F08F8.10		
CEOP3436	T20B12.1, T20B12.3		
CEOP3440	H14A12.3		
CEOP3448	ZK686.2		
CEOP3452	ZK686.4		
CEOP3456	ZK652.9		
CEOP3460	ZK652.3		
CEOP3462	ZK688.9, ZK688.5		
CEOP3464	C29E4.5		
CEOP3465	C29E4.3		
CEOP3466	C29E4.12		
CEOP3468	F44B9.6		

[1]Huang P, Pleasance ED, Maydan JS, Hunt-Newbury R, O'Neil NJ, et al. (2007) Identification and analysis of internal promoters in Caenorhabditis elegans operons. Genome Res 17: 1478-1485.

CEOP3476	K12H4.5		
CEOP3480	K06H7.4		
CEOP3484	D2007.5		
CEOP3488	C30A5.2, C02F5.6, C30A5.3		
CEOP3500	F10E9.3, F10E9.5		
CEOP3504	F10E9.11		
CEOP3508	ZC262.8		
CEOP3512	R05D3.7		
CEOP3516	R05D3.2		
CEOP3520	ZK353.8		
CEOP3528	C30C11.2		
CEOP3536	C06E1.11		
CEOP3538	B0303.15		
CEOP3540	K02D10.1, PAR2.1		
CEOP3544	F44E2.10		
CEOP3548	F44E2.7, F44E2.9		
CEOP3552	ZK637.2		
CEOP3560	R08D7.4		
CEOP3564	F59B2.3		
CEOP3572	R107.6		
CEOP3576	C38C10.4		
CEOP3580	F54C8.4		
CEOP3584	F54C8.5		
CEOP3596	ZK1098.6, ZK1098.1		
CEOP3600	C48B4.7		
CEOP3604	C48B4.10		
CEOP3608	F58A4.9		
CEOP3612	C07A9.2		
CEOP3616	T05G5.6, T05G5.5, T05G5.9		
CEOP3620	R10E11.8		
CEOP3624	ZK632.2, ZK632.1		
CEOP3628	ZK632.3	CEOP3628	ZK632.3
CEOP3632	ZK632.7		
CEOP3636	ZK632.9		
CEOP3640	ZK632.12, K11H3.4		
CEOP3642	K03H1.2		
CEOP3644	T16G12.5		
CEOP3648	T16H12.1		
CEOP3652	T16H12.5		
CEOP3656	ZK1128.1		
CEOP3657	ZK1128.6		
CEOP3660	T20G5.11		
CEOP3664	R01H10.6, R01H10.7	CEOP3664	R01H10.6
CEOP3668	M03C11.2, M03C11.4		
CEOP3672	M03C11.8		
CEOP3676	D2045.9, D2045.6		
CEOP3680	Y45F3A.2		
CEOP3684	Y39A1A.1		
CEOP3692	Y39A1A.13, Y39A1A.12		
CEOP3700	K01G5.8, K01G5.7		
CEOP3701	K01G5.10		
CEOP3702	K01G5.9		
CEOP3708	M142.5		
CEOP3712	Y48A6B.5, Y48A6B.3	CEOP3712	Y48A6B.5
CEOP3716	W09D6.1, Y48A6B.11		
CEOP3720	Y47D3A.29		
CEOP3722	Y47D3A.27		
CEOP3723	Y66D12A.8		
CEOP3728	Y41C4A.10		
CEOP3732	Y56A3A.4		
CEOP3744	Y56A3A.13, Y56A3A.21		
CEOP3746	Y56A3A.30		
CEOP3748	Y56A3A.32, Y56A3A.31		
CEOP3750	Y79H2A.6		

[1]Huang P, Pleasance ED, Maydan JS, Hunt-Newbury R, O'Neil NJ, et al. (2007) Identification and analysis of internal promoters in Caenorhabditis elegans operons. Genome Res 17: 1478-1485.

CEOP3752	Y75B8A.7		
CEOP3756	Y75B8A.18		
CEOP3760	Y49E10.3		
CEOP3784	F53A2.4		
CEOP3792	T27E9.2		
CEOP3800	T27E9.1		
CEOP3804	T28A8.6		
CEOP3820	C54C6.2		
CEOP3830	ZK1098.10		
CEOP3831	C24H11.6		
CEOP3833	B0361.5, B0361.6		
CEOP3834	Y48G9A.1		
CEOP3835	T04A8.16		
CEOP3836	R151.2, R151.3		
CEOP3838	Y82E9BR.18, Y82E9BR.19		
CEOP3839	T07C4.4, T07C4.1		
CEOP3840	T26A5.9		
CEOP3841	Y56A3A.7		
CEOP3842	K01G5.3, K01G5.5		
CEOP3843	Y47D3A.22		
CEOP3844	T07C4.11		
CEOP4004	F29C4.7		
CEOP4007	Y38C1AA.1		
CEOP4008	R02D3.4, R02D3.3, R02D3.5		
CEOP4012	T21D12.3, T21D12.4	CEOP4012	T21D12.3
CEOP4016	Y66H1B.4, T07A9.1		
CEOP4020	T07A9.9		
CEOP4024	T07A9.5, T07A9.6		
CEOP4028	Y66H1A.3		
CEOP4032	K11H12.2		
CEOP4040	Y55F3BL.1, F56B3.5		
CEOP4044	Y55F3AM.6		
CEOP4047	Y41D4B.12, Y41D4B.13		
CEOP4048	Y55F3AR.2		
CEOP4052	Y48A5A.2, F52C12.2, F52C12.4		
CEOP4056	Y76B12C.2	CEOP4056	Y76B12C.2
CEOP4060	Y38F2AR.1, Y38F2AR.2		
CEOP4068	Y94H6A.9		
CEOP4072	Y54G2A.31, Y54G2A.22, Y54G2A.23		
CEOP4076	Y67D8A.1		
CEOP4084	B0546.4		
CEOP4092	M57.2, M57.1, B0212.3		
CEOP4096	Y37E11B.1		
CEOP4100	Y37E11B.6		
CEOP4104	Y37E11AM.3, Y37E11AM.2		
CEOP4112	C35B1.2		
CEOP4116	K08D10.12, K08D10.4		
CEOP4120	Y24D9A.8, F55F10.1		
CEOP4124	ZK180.4		
CEOP4128	F29B9.4, F29B9.6		
CEOP4132	F29B9.11		
CEOP4136	F29B9.2		
CEOP4140	K08F11.4, K08F11.5		
CEOP4144	E04A4.4, E04A4.5		
CEOP4148	E04A4.6		
CEOP4152	R11E3.8, R11E3.6, H06H21.6	CEOP4152	R11E3.8
CEOP4156	W03F8.4, W03F8.3, W03F8.5		
CEOP4160	F41H10.11		
CEOP4168	T12E12.2, T12E12.4		
CEOP4172	T19E7.3		
CEOP4174	C11D2.4		
CEOP4176	Y73B6BL.6, Y73B6BL.32	CEOP4176	
CEOP4180	C01G5.5, C01G5.6		
CEOP4184	F38A5.1, F38A5.2		

[1]Huang P, Pleasance ED, Maydan JS, Hunt-Newbury R, O'Neil NJ, et al. (2007) Identification and analysis of internal promoters in Caenorhabditis elegans operons. Genome Res 17: 1478-1485.

CEOP4188	C01B10.9		
CEOP4192	Y73B6A.5		
CEOP4196	H34C03.2		
CEOP4204	T22D1.3, T22D1.4		
CEOP4208	C06G3.10		
CEOP4212	C34D4.12, C34D4.14		
CEOP4216	D2024.6		
CEOP4228	F49E8.5		
CEOP4232	F32E10.5		
CEOP4236	F45E4.10		
CEOP4240	F33D4.4, F33D4.7		
CEOP4244	C46A5.6		
CEOP4248	C33H5.7		
CEOP4252	C33H5.11		
CEOP4256	C33H5.14, C33H5.15		
CEOP4260	C33H5.17, C33H5.18		
CEOP4264	C49H3.5		
CEOP4268	C26B2.7		
CEOP4272	F42G8.3, F42G8.5		
CEOP4274	C07G1.4, C07G1.5		
CEOP4276	K07H8.9, K07H8.10		
CEOP4280	F35H10.7, K07H8.3		
CEOP4284	D2096.12, D2096.4		
CEOP4288	D2096.7, D2096.8		
CEOP4292	C28C12.12, C28C12.9		
CEOP4294	C09G4.3, C09G4.5		
CEOP4296	F08B4.6		
CEOP4300	F21D5.2, F21D5.1	CEOP4300	F21D5.2
CEOP4304	F21D5.5, F21D5.7		
CEOP4308	C33D9.5		
CEOP4310	C09G9.1, C09G9.2		
CEOP4312	C27B7.2		
CEOP4320	C53B4.6, C53B4.4		
CEOP4324	C01F6.9		
CEOP4328	T20D3.7		
CEOP4336	C28D4.9		
CEOP4340	K07F5.13		
CEOP4344	C47E12.3		
CEOP4348	C47E12.5, C47E12.4	CEOP4348	C47E12.5
CEOP4352	K08F4.1, C04G2.11		
CEOP4356	T14G10.7, T14G10.6		
CEOP4360	K04D7.1		
CEOP4368	Y43C5A.6		
CEOP4372	R11A8.5		
CEOP4376	R11A8.7		
CEOP4380	R10H10.7, F13B12.6		
CEOP4384	T07G12.8, T07G12.12, T07G12.6		
CEOP4392	R102.5		
CEOP4396	T11G6.1		
CEOP4400	F13H10.3, F13H10.2, F13H10.4		
CEOP4404	M7.1		
CEOP4408	T05E11.6, T05E11.1		
CEOP4412	F01G4.1		
CEOP4420	C08F8.2		
CEOP4424	R07H5.2		
CEOP4428	B0035.15		
CEOP4432	B0035.3		
CEOP4436	B0035.6		
CEOP4440	B0035.12		
CEOP4448	M04B2.1		
CEOP4452	F12F6.8, F12F6.7, F12F6.6		
CEOP4460	F58B3.4, F58B3.5		
CEOP4464	F58B3.7, F58B3.6		
CEOP4468	ZK792.1, ZK809.5		

[1]Huang P, Pleasance ED, Maydan JS, Hunt-Newbury R, O'Neil NJ, et al. (2007) Identification and analysis of internal promoters in Caenorhabditis elegans operons. Genome Res 17: 1478-1485.

CEOP4472	ZK792.6		
CEOP4480	F38H4.9		
CEOP4484	ZK829.7		
CEOP4488	F11A10.1		
CEOP4490	F11A10.6, F11A10.8		
CEOP4492	M18.3, F11A10.5		
CEOP4496	M18.7		
CEOP4500	B0001.3, B0001.2, B0001.7		
CEOP4504	C42C1.13		
CEOP4508	C42C1.9, C42C1.16, C42C1.10		
CEOP4512	F19B6.2, F19B6.1		
CEOP4516	C25G4.5		
CEOP4520	K08E7.1, K08E7.2	CEOP4520	K08E7.1
CEOP4524	Y55D9A.2, Y55D9A.1		
CEOP4532	B0564.7, B0564.1		
CEOP4535	JC8.13		
CEOP4536	JC8.5, JC8.6		
CEOP4538	Y67H2A.7, Y67H2A.4		
CEOP4539	Y67H2A.10		
CEOP4540	Y62E10A.6, Y62E10A.2		
CEOP4544	Y62E10A.9		
CEOP4548	Y62E10A.12		
CEOP4552	K09B11.1		
CEOP4556	F52G2.1, F52G2.2		
CEOP4560	Y45F10D.12		
CEOP4568	B0513.3		
CEOP4572	Y37A1B.15		
CEOP4576	F52B11.2, F52B11.1, Y37A1B.1		
CEOP4580	Y57G11C.11	CEOP4580	Y57G11C.11
CEOP4581	Y41E3.11, Y41E3.10, Y41E3.9		
CEOP4582	Y73F8A.25		
CEOP4588	Y116A8A.9, T06A10.1		
CEOP4590	T06A10.3		
CEOP4592	Y116A8C.9		
CEOP4596	Y116A8C.30, Y116A8C.28, Y116A8C.35		
CEOP4600	ZK550.3		
CEOP4608	C30H6.7		
CEOP4612	C42C1.4		
CEOP4616	F42A9.1		
CEOP4622	T07A9.11		
CEOP4624	T13F2.2		
CEOP4626	C27D8.4, T23B5.3		
CEOP4630	Y104H12D.2		
CEOP4632	Y116A8C.26		
CEOP4634	Y17G9B.2		
CEOP4636	Y38C1AA.2, Y38C1AA.12		
CEOP4638	Y38F2AL.4		
CEOP4640	Y41D4B.4, Y41D4B.5		
CEOP4642	Y55F3AM.9		
CEOP4644	Y57G11C.36		
CEOP4646	Y67D8C.3, Y67D8C.4		
CEOP4647	F15B10.2, F15B10.1		
CEOP4648	M70.5		
CEOP4649	Y73B6BL.18		
CEOP4651	Y57G11C.34		
CEOP4652	C46G7.2		
CEOP4653	Y37E11AL.7		
CEOP4654	T23B5.4		
CEOP4655	Y71G10AL.1		
CEOP5004	Y38C9A.2		
CEOP5006	W03F9.5		
CEOP5008	DC2.3		
CEOP5012	F33E11.3, F33E11.6		
CEOP5016	T22H9.2		

[1]Huang P, Pleasance ED, Maydan JS, Hunt-Newbury R, O'Neil NJ, et al. (2007) Identification and analysis of internal promoters in Caenorhabditis elegans operons. Genome Res 17: 1478-1485.

CEOP5026	Y50D4A.1, Y50D4A.5		
CEOP5032	Y45G12B.1, Y45G12B.2		
CEOP5048	H43I07.3		
CEOP5052	F32D1.1		
CEOP5056	F32D1.6		
CEOP5060	K03B4.3		
CEOP5064	R02F11.4, C37H5.6		
CEOP5072	C04F5.9		
CEOP5076	CD4.4		
CEOP5080	F25B4.6		
CEOP5084	F44E7.9		
CEOP5092	M03F8.2, M03F8.3		
CEOP5100	R01B10.6		
CEOP5104	Y38A10A.7		
CEOP5108	T05H4.13		
CEOP5118	W01A11.1, W01A11.2	CEOP5118	W01A11.1
CEOP5120	F46E10.9, F46E10.8, F46E10.10		
CEOP5128	T10H9.4		
CEOP5132	W02F12.5, W02F12.4, W02F12.6		
CEOP5140	F09G2.8, F09G2.9		
CEOP5144	C13F10.6		
CEOP5152	C05C8.9		
CEOP5164	C26F1.4		
CEOP5168	C37C3.2		
CEOP5172	Y97E10AR.4, Y97E10AR.6	CEOP5172	
CEOP5176	ZC513.5, ZC513.4		
CEOP5180	D1014.4		
CEOP5184	W02D7.6		
CEOP5188	T23B12.7		
CEOP5200	F25G6.9		
CEOP5220	T18H9.6, T18H9.7		
CEOP5224	K07B1.3		
CEOP5228	F36D4.2, F36D4.3		
CEOP5232	C50F4.12, C50F4.11		
CEOP5236	F46B6.6, F46B6.4, F46B6.3, F46B6.7		
CEOP5240	K08H10.7		
CEOP5244	C08B6.7		
CEOP5246	ZK856.1		
CEOP5248	ZK856.13, ZK856.11, ZK856.9		
CEOP5252	B0024.10, B0024.11		
CEOP5254	K07C5.3, K07C5.2		
CEOP5256	T21C9.4		
CEOP5260	D1054.2		
CEOP5264	D1054.13, D1054.15		
CEOP5268	F17C11.8, F17C11.10		
CEOP5276	C06H2.6		
CEOP5280	T19B10.7		
CEOP5288	C55A6.10		
CEOP5296	T27F2.3		
CEOP5300	F55A11.2		
CEOP5308	C52E4.3		
CEOP5312	F57F5.5, F10C2.2		
CEOP5316	F10C2.6, F10C2.4		
CEOP5321	R07B7.4		
CEOP5324	ZK863.4		
CEOP5328	ZK863.6		
CEOP5336	T04H1.5, T04H1.4		
CEOP5340	F55C5.7, F55C5.8		
CEOP5344	R04F11.5		
CEOP5348	W04D2.4		
CEOP5356	F45D3.5		
CEOP5360	C14C10.3		
CEOP5364	K10C8.3		
CEOP5368	R11D1.10		

[1]Huang P, Pleasance ED, Maydan JS, Hunt-Newbury R, O'Neil NJ, et al. (2007) Identification and analysis of internal promoters in Caenorhabditis elegans operons. Genome Res 17: 1478-1485.

CEOP5372	C15C8.4		
CEOP5376	T16G1.10		
CEOP5384	T09E8.2		
CEOP5385	C50B6.8		
CEOP5388	F53F1.3, F53F1.2		
CEOP5392	F53F4.10		
CEOP5396	F53F4.12		
CEOP5398	F58G11.2, F58G11.1		
CEOP5400	F53C11.8		
CEOP5404	F55B12.3		
CEOP5412	Y49A3A.5, Y49A3A.1	CEOP5412	
CEOP5416	C15H11.4, C15H11.9		
CEOP5420	F23B12.6		
CEOP5424	C53A5.2, C53A5.1		
CEOP5432	T01C3.8		
CEOP5436	T01C3.10, F14H8.1		
CEOP5440	C25D7.10, C25D7.8		
CEOP5444	F28F8.6		
CEOP5452	T10C6.6, T10C6.5		
CEOP5454	Y32B12B.2		
CEOP5456	W06H3.3		
CEOP5460	F59A1.9, F59A1.7		
CEOP5464	Y59A8B.6		
CEOP5472	Y80D3A.2, Y80D3A.4		
CEOP5476	Y39B6A.43		
CEOP5480	Y39B6A.36, Y39B6A.34	CEOP5480	
CEOP5488	Y39B6A.13, Y39B6A.12		
CEOP5496	Y60A3A.13, Y60A3A.12		
CEOP5500	Y113G7A.9		
CEOP5504	Y113G7B.7		
CEOP5508	Y113G7B.23, Y113G7B.18		
CEOP5516	T03D8.2, F31D4.1, T03D8.1		
CEOP5518	F23H12.2		
CEOP5522	F47G9.1		
CEOP5524	F55B12.5		
CEOP5526	R186.8		
CEOP5530	H27A22.1, T21C9.12		
CEOP5532	W05B10.2		
CEOP5534	Y57E12AL.1		
CEOP5537	F32D8.6		
CEOP5538	W08G11.3, W08G11.4		
CEOP5541	R31.2, R31.1		
CEOP5542	Y60A3A.10, Y60A3A.9		
CEOP5543	F22F7.1		
CEOPX004	T08D2.5, T08D2.4, T08D2.3		
CEOPX008	F35H12.3		
CEOPX012	F53H8.1		
CEOPX016	W05H7.4		
CEOPX024	T26C11.6, T26C11.7		
CEOPX040	C15C7.2		
CEOPX048	F55D10.3		
CEOPX052	H28G03.2		
CEOPX060	C26B9.1		
CEOPX068	R07E4.4		
CEOPX072	T13C5.6		
CEOPX076	C15B12.7		
CEOPX080	K11G12.6		
CEOPX084	F41B4.3		
CEOPX092	C14F5.5		
CEOPX100	F08F1.7		
CEOPX107	ZC506.1		
CEOPX108	F45E6.2		
CEOPX109	T20B5.1		
CEOPX112	F11C1.1		

[1]Huang P, Pleasance ED, Maydan JS, Hunt-Newbury R, O'Neil NJ, et al. (2007) Identification and analysis of internal promoters in Caenorhabditis elegans operons. Genome Res 17: 1478-1485.

CEOPX116	R12H7.3		
CEOPX126	F48F7.6, F48F7.5		
CEOPX128	R03E1.1		
CEOPX130	F20D1.3, F20D1.2	CEOPX130	
CEOPX132	C18B12.6		
CEOPX134	C11H1.3		
CEOPX136	H13N06.3		
CEOPX140	ZK662.4		
CEOPX144	T25G12.5		
CEOPX156	K09E9.3, K09E9.2		
CEOPX164	W05H9.4		
CEOPX166	Y34B4A.2		
CEOPX167	C55B6.2		
CEOPX172	F14B8.2		

[1]Huang P, Pleasance ED, Maydan JS, Hunt-Newbury R, O'Neil NJ, et al. (2007) Identification and analysis of internal promoters in Caenorhabditis elegans operons. Genome Res 17: 1478-1485.
