# Supplementary material for: The Genomic Distribution and Function of Histone Variant HTZ-1 during C. elegans Embryogenesis
Source: PLoS Genet. 2008 Sep 12;4(9):e1000187. doi: 10.1371/journal.pgen.1000187 (PMC2522285; doi:10.1371/journal.pgen.1000187)
Supplement: Text S1 — Supplemental text. (0.12 MB PDF) [file pgen.1000187.s012.pdf]

TEXT S1

Notes on temporal effects on ChIP and transcription analysis

The analysis correlating our ChIP data with transcript levels should be interpreted with the following in mind. Our experiments were performed in a temporally mixed population of whole embryos, each of which contains diverse cell types. Therefore, our reported peaks may represent occupancy that occurs across cell types or temporal stages in the population. For that reason, our data is not directly comparable to any single time-point of the published developmental gene expression time-course, which was temporally synchronized [1]. The last three timepoints of the transcription study (122, 143, and 186 minutes) correspond most closely to the population structure of our sample [1]. For that reason, we averaged transcript levels recorded at these three time points to make correlations with the RNA polymerase occupancy in our mixed population. Our conclusions using this strategy were confirmed using an independent published expression dataset derived from a mixed-age population of embryos (Figure S3) [2].

RNAi phenotype details

In about 5% of arrested RNAi-treated embryos we observed chromosome segregation defects (data not shown). However, this phenotype was not observed in arrested embryos harbored by maternally rescued *htz-1(tm2469)* homozygous animals. It is unclear why chromosome segregation defects were observed only in the *htz-1* RNAi animals, since arrested embryos born of *htz-1(tm2469)* homozygous mothers should also lack any maternal contribution of HTZ-1 RNA or protein. Perhaps relevant to our study, a similar lagging chromosome phenotype was observed in mammalian cells subjected to HTZ-1 siRNA, but was not reported for the homozygous mutants [3].

Previously identified sites of cell specific HTZ-1 activity

HTZ-1 was shown to affect two PHA-4 dependent promoters, *myo-2* and *R07B1.9* [4]. Our ChIP-chip data did not detect HTZ-1 incorporation at those loci. One explanation is rooted in the fact that our data was derived from a mixed population of whole embryos, whereas the association of HTZ-1 with these promoters occurs transiently and in a subset of cells, which may have caused them to fall below our detection limit. Consistent with this explanation, we did not observe RNA polymerase II enrichment at these promoters, even though the genes are known to be activated during embryogenesis.

Notes on attempts of zygotic HTZ-1 rescue

We attempted to determine whether the maternal contribution is strictly necessary for embryogenesis, or whether zygotically produced HTZ-1 protein could suffice. Strong evidence that the maternal contribution is specifically necessary for embryogenesis is given by the lack of viable cross-progeny from attempts to create an M^-Z^+ embryo by mating an M^+Z^- mother with a wild-type male. However, any defects in the vulva or oocytes of the M^+Z^- mother could prevent insemination or fertilization, so the lack of offspring is not conclusive.

SUPPLEMENTAL FIGURE AND TABLE LEGENDS

Table S1. Complete list of all over-represented Gene Ontology Terms. A table with the complete list of GO terms overrepresented among HTZ-1 bound genes. A Bonferroni-corrected p-value of >0.01 was used. The number of genes annotated to the term in the HTZ-1 bound set and the entire genome are indicated, as are GO term definitions.

Table S2. The relationship between operons, operon genes, and HTZ-1 occupancy at promoters. A table listing all operons and operon genes found to contain an HTZ-1 peak. Operons and genes found to possess internal promoters in Huang *et al.* are noted.

Table S3. HTZ-1 peak calls. A table listing the genome coordinates of 5164 HTZ-1 ChIPOTLe peaks, associated ChIPOTLe p-values, and nearby annotated genes. All coordinates and annotations are based on genome release ws120, March 2004.

Figure S1. Anti-HTZ-1 antibody recognizes a single ~15kD protein. A western blot showing mixed stage N2 worms (lane 1) or N2 embryos (lane 2) detecting using anti-HTZ-1 antibody to the C-terminal peptide PGKPGAPGQGPQ (amino acids 128-140). The predicted size of HTZ-1 is 14.7 kD.

Figure S2. Internal promoters identified by HTZ-1 occupancy are differentially expressed during early embryogenesis. A published time-course of transcript levels during early embryogenesis [1] is plotted for genes in operons (A) CEOP1120, (B) CEOP4008), and (C) CEOPX024. The left panels show the operon loci; operon genes with peaks of HTZ-1 upstream are denoted by the asterisks(*).

Figure S3. HTZ-1 occupancy at promoters is positively correlated with embryo expression. Genes were sorted by $\log_2$(embryo RNA/mixed reference RNA) and used to calculate a moving average of HTZ-1 ChIP enrichment z-scores (y-axis; window=100, step=1). HTZ-1 incorporation at each gene was scored by averaging all probes within 1 kb of the translation start site. The reported embryo expression values are derived from a published embryo expression dataset [2].

Figure S4. Anti-HTZ-1 C-terminal antibody is not specifically excluded from the X chromosome. HTZ-1::YFP embryo co-stained with **(A)** DAPI, **(B)** anti-YFP antibody, **(C)** anti-DPY-27 antibody. **(D)** Merged DPY-27 (red) and YFP (green). One cell is outlined and enlarged 3X to highlight the distinction between HTZ-1 and YFP staining (lower left).

Figure S5. HTZ-1 occupancy at the promoters of known dosage compensated genes *apl-1* and *lin-15A/B*. (A) A genome browser view of the *lin-15A/B* locus on the X chromosome. Z-scores (calculated from the $\log_2$ ratio (ChIP/Input)) for the median of four HTZ-1 ChIPs, median of three DPY-27 ChIPs [5], mean of two RNA Polymerase II ChIPs and median of three no antibody ChIPs are plotted. Peaks of HTZ-1 binding are denoted, with gene annotations indicated below. The blue arrows specify the direction of transcription. The CEOPX140 operon is indicated by the black arrow. **(B)** Same as (A), except at the *apl-1* (previously named *uvt-4)* locus.

Figure S6. *C. elegans* HTZ-1 is more similar to *Drosophila* H2Avd and vertebrate H2A.Z than yeast Htz1. A CLUSTALW [6] alignment of 5 species H2A.Z homologues in shown is

visualized in Jalview [7]. Shading is by percent identity with dark blue = 100 %ID, medium blue >80 %ID, light blue > 60%ID.

Figure S7. HTZ-1 peaks are coincident with DPY-27 peaks on the X chromosome. Locations of HTZ-1 incorporation were compared with DPY-27 peak distribution [5]. The Venn diagrams display the coincidence between the 495 HTZ-1 peaks and 1499 DPY-27 peaks on X with a **(A)** 1000-bp pverlap window or **(B)** 10-bp overlap window.

Figure S8. A comparison of mean HTZ-1 peak height and width between X and the autosomes. A histogram of HTZ-1 ChIPOTLe peak **(A)** width (bp) and **(B)** height (mean z-score) is shown for 495 X chromosome peaks (blue) and 4669 autosome peaks (pink). Each bin is plotted as percent of class.

References

1. Baugh LR, Hill AA, Slonim DK, Brown EL, Hunter CP (2003) Composition and dynamics of the Caenorhabditis elegans early embryonic transcriptome. Development 130: 889-900.
2. Jiang M, Ryu J, Kiraly M, Duke K, Reinke V, et al. (2001) Genome-wide analysis of developmental and sex-regulated gene expression profiles in Caenorhabditis elegans. Proc Natl Acad Sci U S A 98: 218-223.
3. Rangasamy D, Greaves I, Tremethick DJ (2004) RNA interference demonstrates a novel role for H2A.Z in chromosome segregation. Nat Struct Mol Biol 11: 650-655.
4. Updike DL, Mango SE (2006) Temporal regulation of foregut development by HTZ-1/H2A.Z and PHA-4/FoxA. PLoS Genet 2: e161.
5. Ercan S, Giresi PG, Whittle CM, Zhang X, Green RD, et al. (2007) X chromosome repression by localization of the C. elegans dosage compensation machinery to sites of transcription initiation. Nat Genet 39: 403-408.
6. Larkin MA, Blackshields G, Brown NP, Chenna R, McGettigan PA, et al. (2007) Clustal W and Clustal X version 2.0. Bioinformatics 23: 2947-2948.
7. Clamp M, Cuff J, Searle SM, Barton GJ (2004) The Jalview Java alignment editor. Bioinformatics 20: 426-427.
